# Accelerating supercritical pharmaceutical formulation via interpretable data-driven prediction of drug solubility

**DOI:** 10.1038/s41598-026-44161-9

**Published:** 2026-03-14

**Authors:** El-Sayed Khafagy, Amr Selim Abu Lila, Mahboubeh Pishnamazi

**Affiliations:** 1 Department of Pharmaceutics, College of Pharmacy, Prince Sattam bin Abdulaziz University, Al-kharj, 11942 Saudi Arabia; 2 Department of Pharmaceutics, College of Pharmacy, University of Ha’il, Ha’il, 81442 Saudi Arabia; 3 Institute of Research and Development, Duy Tan University, Da Nang, Vietnam; 4 School of Engineering & Technology, Duy Tan University, Da Nang, Vietnam

**Keywords:** Pharmaceutical Formulation, Drug Solubility Modeling, Machine Learning in Pharmaceutics, Data-Driven Drug Design, Chemistry, Computational biology and bioinformatics, Drug discovery, Mathematics and computing

## Abstract

Drug solubility in supercritical carbon dioxide (SC-CO_2_) plays a pivotal role in the development of particle engineering, drug loading, and solvent-free pharmaceutical formulations. However, experimental solubility determination in supercritical systems remains costly, time-consuming, and compound-specific. In this study, an interpretable data-driven framework is proposed to support pharmaceutical formulation scientists by accurately predicting drug solubility in SC-CO_2_ while elucidating the governing physicochemical factors. Multiple machine learning regressors, including Extreme Gradient Boosting and Support Vector Regression, were developed and further integrated into an ensemble strategy to enhance robustness and generalizability. Model performance was systematically optimized using bio-inspired metaheuristic algorithms, enabling efficient hyperparameter selection across complex, nonlinear search spaces. Beyond predictive accuracy, model interpretability was emphasized through sensitivity-based and amplitude-based feature analyses, revealing the dominant molecular descriptors and process conditions influencing solubility behavior. The results demonstrate that the proposed framework not only improves solubility prediction accuracy but also provides mechanistic insights relevant to drug selection, formulation feasibility, and supercritical processing design. This work establishes a practical computational tool for accelerating pharmaceutical development pipelines involving supercritical fluid technologies.

**Supplementary Information:**

The online version contains supplementary material available at 10.1038/s41598-026-44161-9.

## Introduction

The solubility of pharmaceutical compounds strongly influences their absorption, bioavailability, and therapeutic performance. Many modern drugs suffer from poor aqueous solubility, complicating formulation and requiring extensive experimental optimization^1,2^. Traditional improvement strategies such as salt formation, particle size reduction, and cosolvent use are often labor-intensive and may introduce stability or safety challenges, motivating the search for more efficient solubility-enhancing approaches^3–5^. Supercritical fluids (SCFs) have emerged as promising alternatives due to their tunable physicochemical properties^6–8^. Above their critical point, SCFs exhibit liquid-like solvating power and gas-like diffusivity, enabling rapid mass transfer and controllable solvent strength^9,10^. Supercritical carbon dioxide (SC-CO_2_) is particularly attractive owing to its non-toxicity, environmental compatibility, mild critical conditions, and easy removal from final products^11^. Its solvating capacity can be finely adjusted through pressure and temperature, allowing selective solubilization of diverse drug molecules^12,13^.

Solubility in SC-CO_2_ depends on the combined effects of drug molecular characteristics such as polarity, hydrogen-bonding ability, and molecular weight and process variables including solvent density and operating conditions^14^. Pressure typically increases solubility by raising solvent density, whereas temperature effects are nonlinear due to competing influences on density and vapor pressure^15^. Mapping these interactions experimentally is resource-intensive and may not fully capture the complexity of multidimensional solubility behavior^16,17^. Beyond solubility enhancement, SCFs enable particle engineering and micronization, improving dissolution rates and facilitating controlled drug release^18,19^. However, accurately predicting solubility across chemically diverse compounds remains challenging, reinforcing the need for reliable predictive frameworks to minimize trial-and-error experimentation and accelerate formulation development^20–23^.

Zhu et al.^24^ developed an Adaptive Neuro-Fuzzy Inference System (ANFIS) to predict the solubility of busulfan, an anticancer drug, in supercritical carbon dioxide. The ANFIS structure, built with Trimf membership functions and Grid Partitioning, demonstrated high accuracy. The findings highlighted ANFIS as a reliable tool for modeling drug solubility, supporting supercritical technology for improved pharmaceutical formulations. Najmi et al.^25^ investigated the solubility of five drugs, Niflumic acid, Tolfenamic acid, Glibenclamide, Nystatin, and Rivaroxaban, in SC-CO_2_ under different pressure and temperature conditions. To establish a reliable predictive framework, models include Polynomial Regression (PR), Multilayer Perceptron (MLP), and K-Nearest Neighbors (KNN), which were applied, with their parameters optimized using the Harmony Search (HS) algorithm. Among the tested approaches, HS-PR achieved the highest accuracy with an R^2^ value of 0.96449, demonstrating strong predictive capability for drug solubility. Alotaibi et al.^26^ addressed the challenge of poor water solubility in new medications by applying supercritical processing to predict raloxifene solubility and supercritical CO_2_ density using temperature and pressure as inputs. Three regression models, Extra Trees (ET), Random Forest (RF), and Gradient Boosting (GB), were optimized with gradient-based techniques for accurate prediction. GB achieved the best performance for density with an R^2^ of 0.986 and an RMSE of 23.20, while ET provided the most accurate solubility prediction with an R^2^ of 0.949 and an RMSE of 0.41. Damansabz et al.^27^ developed predictive models for compound solubility in supercritical CO_2_ using temperature, pressure, and density as inputs. XGBoost, deep learning, and a novel hybrid GWO-DE-LSTM model were evaluated. XGBoost achieved R^2^ = 0.881 with balanced feature use, while deep learning reached R^2^ = 0.895 with strong dependence on pressure. The hybrid GWO-DE-LSTM outperformed others (R^2^=0.931), emphasizing temperature as the key factor. Findings showed that evolutionary hybridization improved prediction accuracy and revealed distinct feature–solubility relationships. Bahrami et al.^28^ focused on accurately predicting the solubility of solid drugs (SDs) in SC-CO_2_ using artificial intelligence models, specifically Gene Expression Programming (GEP) and ANFIS. The ANFIS model outperformed GEP, achieving R^2^ values of 0.991 (training) and 0.990 (validation) with low errors. Sensitivity analysis revealed molecular weight, pressure, melting point, and temperature as key factors, with molecular weight being the most influential. Alsaab and Althobaiti^29^ investigated the prediction of phenytoin solubility in supercritical conditions using machine learning (ML) techniques to enhance solubility and bioavailability. Temperature, pressure, solubility, and solvent density data were used to train three models: Linear Regression (LR), Gaussian Process Regression (GPR), and Automatic Relevance Determination Regression (ARD). The ADABOOST ensemble method and Jellyfish Optimization algorithm were applied to improve performance. Among the models, ADA-GPR achieved the highest accuracy for solubility (R^2^ = 0.99644) and solvent density (R^2^ = 0.9933), outperforming ADA-LR and ADA-ARD.

Despite recent advances in modeling drug solubility in supercritical carbon dioxide, several limitations persist in the existing literature. Most previous studies have focused on a limited number of compounds or narrow operating ranges, which restricts model generalizability. In addition, many investigations rely on single machine learning algorithms without systematic hyperparameter optimization, and only a few studies provide insight into the relative importance of input variables or the physical interpretation of model predictions. These shortcomings limit the practical applicability of data-driven approaches for formulation and process design in supercritical CO_2_ systems.

**Fig. 1:**
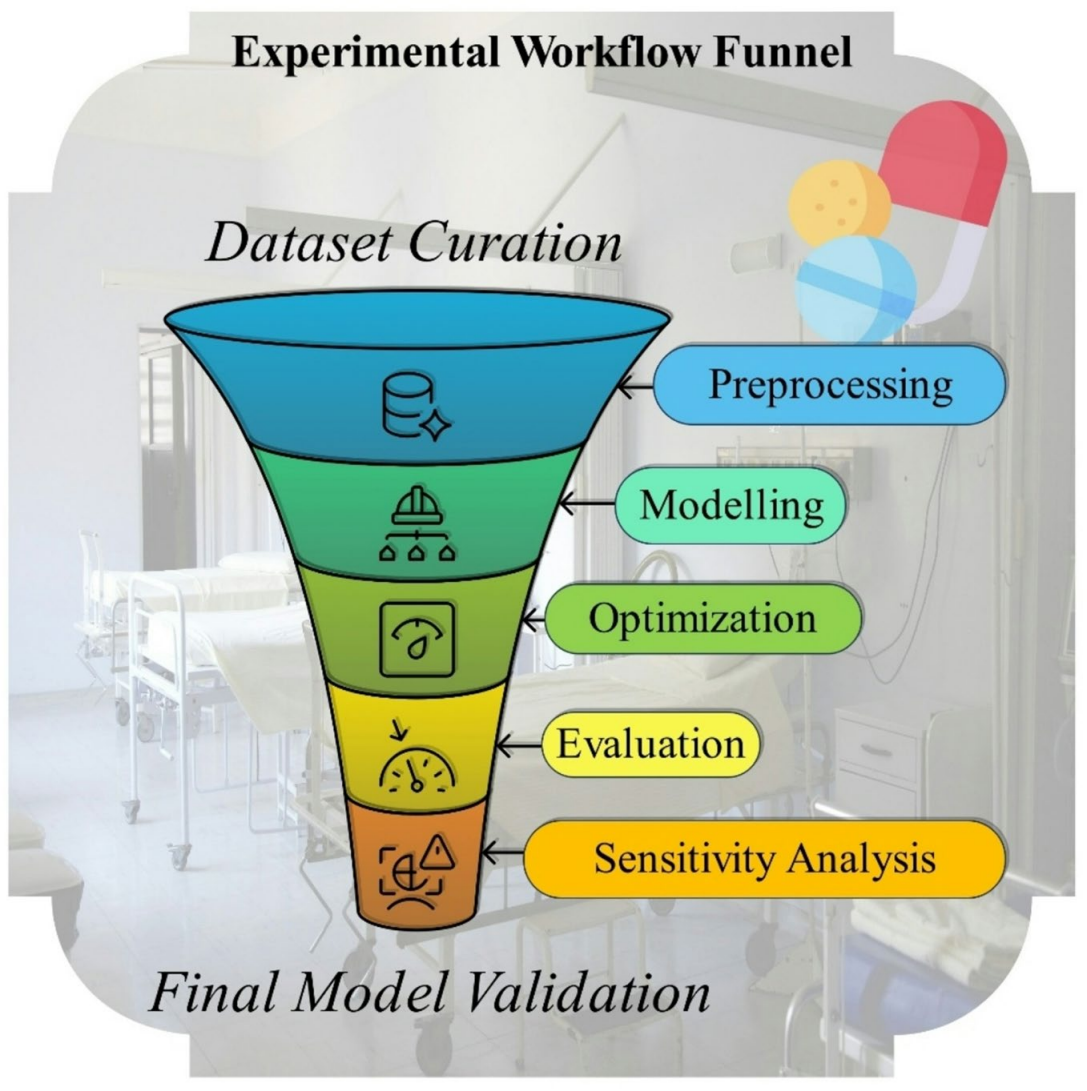
Overall workflow of the study.

This study presents a unified and comparative modeling framework for solubility prediction in supercritical CO_2_ across multiple pharmaceutical compounds. The dataset is compiled from independent experimental studies and includes seven chemically diverse drugs under varying thermodynamic conditions, requiring models that generalize beyond compound-specific trends. The primary contribution lies in the systematic evaluation of baseline learners, optimizer-driven hybrid models, and a non-hybrid ensemble within a unified multi-objective optimization scheme. This structure enables direct assessment of how different model classes respond to bio-inspired optimization and reveals optimizer sensitivity that is masked when only a single optimized architecture is reported. In contrast to prior studies that focus primarily on maximizing predictive accuracy for a given dataset, the present framework emphasizes robustness across chemically distinct systems and consistency with thermodynamic solubility theory. Furthermore, the interpretability analysis is not treated as a purely statistical ranking of features. Instead, model-derived importance patterns are explicitly linked to established molecular and thermodynamic mechanisms, including density-driven solvation, vapor pressure effects, and lattice energy barriers. This physically grounded interpretation supports the use of the proposed framework for formulation-oriented decision-making rather than only numerical prediction. Figure 1 presents the overall procedure of the study.

## Dataset and materials

The study utilizes a curated dataset comprising 252 solubility samples for seven pharmacologically important drugs: famotidine, erlotinib, clonazepam, nystatin, gemifloxacin, favipiravir, and montelukast^30–35^. These compounds represent diverse therapeutic categories (e.g., anti-ulcer, anticancer, antiepileptic, antifungal, antibacterial, antiviral, and anti-asthmatic), ensuring that the dataset spans a broad chemical and therapeutic spectrum. This diversity increases the generalizability of predictive models compared to studies focused on a single class of molecules. Each data entry integrates both molecular descriptors and thermodynamic conditions, enabling a more comprehensive characterization of solubility behavior:

- *Temperature (K)* Captures kinetic influences on dissolution.
- *Molecular Weight (g/mol)* Reflects molecular size and mass effects.
- *Melting Point (°C)* Relates to lattice stability and crystal energy barriers.
- *Pressure (MPa)* Accounts for solute–solvent interactions under non-standard conditions.

The target variable is experimental solubility (g/L × 10), measured under the above conditions. Descriptive statistics for the dataset are summarized in Table 1, showing balanced variability across features and the target.

The solubility dataset used in this study was compiled from six published experimental works (Refs^30–35^. These studies measured the solubility of various drugs in supercritical CO_2_ under equilibrium conditions, using static or semi-static high-pressure cells (typically 12–30 MPa and 308–338 K). For example, Ardestani et al.^31^ reported famotidine solubility in sc‑CO_2_ over this temperature/pressure range with precise control of equilibrium (± 0.1 K in temperature) and iterative sampling until steady-state was confirmed. Bazaei et al.^30^ conducted measurements for erlotinib hydrochloride, maintaining system equilibration for 60 min and collecting replicates to ensure reproducibility. Similarly, the work on clonazepam included repeated runs at each condition to confirm that solubility values were independent of sampling time^32^. For systems with cosolvents (such as the study on favipiravir and montelukast with ethanol), the authors reported clear protocols for adding the cosolvent, equilibration duration, and use of multiple pressure–temperature points to assess reproducibility^35^.

As mentioned, subset of these measurements was obtained in the presence of cosolvents, primarily ethanol. However, cosolvent type and concentration were not consistently reported across all sources and therefore could not be introduced as an independent input variable without introducing artificial bias or missing-value imputation. Consequently, cosolvent presence is treated as part of the intrinsic experimental variability of the dataset. This heterogeneity is partially mitigated through the use of ensemble-based modelling and multi-objective optimization, which are less sensitive to noise and outlying values than single-model approaches. While this strategy enables learning from limited and heterogeneous data, the inclusion of cosolvent descriptors is expected to further improve reproducibility once sufficiently standardized datasets become available. Accordingly, the absence of cosolvent features is identified as a limitation of the present study and a priority direction for future work.

By incorporating these experimentally validated data, the ML models are grounded on robust, well-characterized measurements. The high quality and reproducibility of the source data enhance the reliability of the predictive framework, making it suitable for formulation design and translational application.

**Table 1:** Overview of input features and output variables with their statistical properties.

Features	Unit	Statistical properties			
		Min	Max	Median	Mean
Temperature	(K)	308	338	323	323
Molecular Weight	(g/mol)	157.1	926.09	385.37	427.18
Melting point	(C)	146	239	190	188.38
Pressure	(MPa)	12	30	21	21
Solubility	(g/l) x 10	0.01	9.05	1.06	1.71

**Fig. 2:**
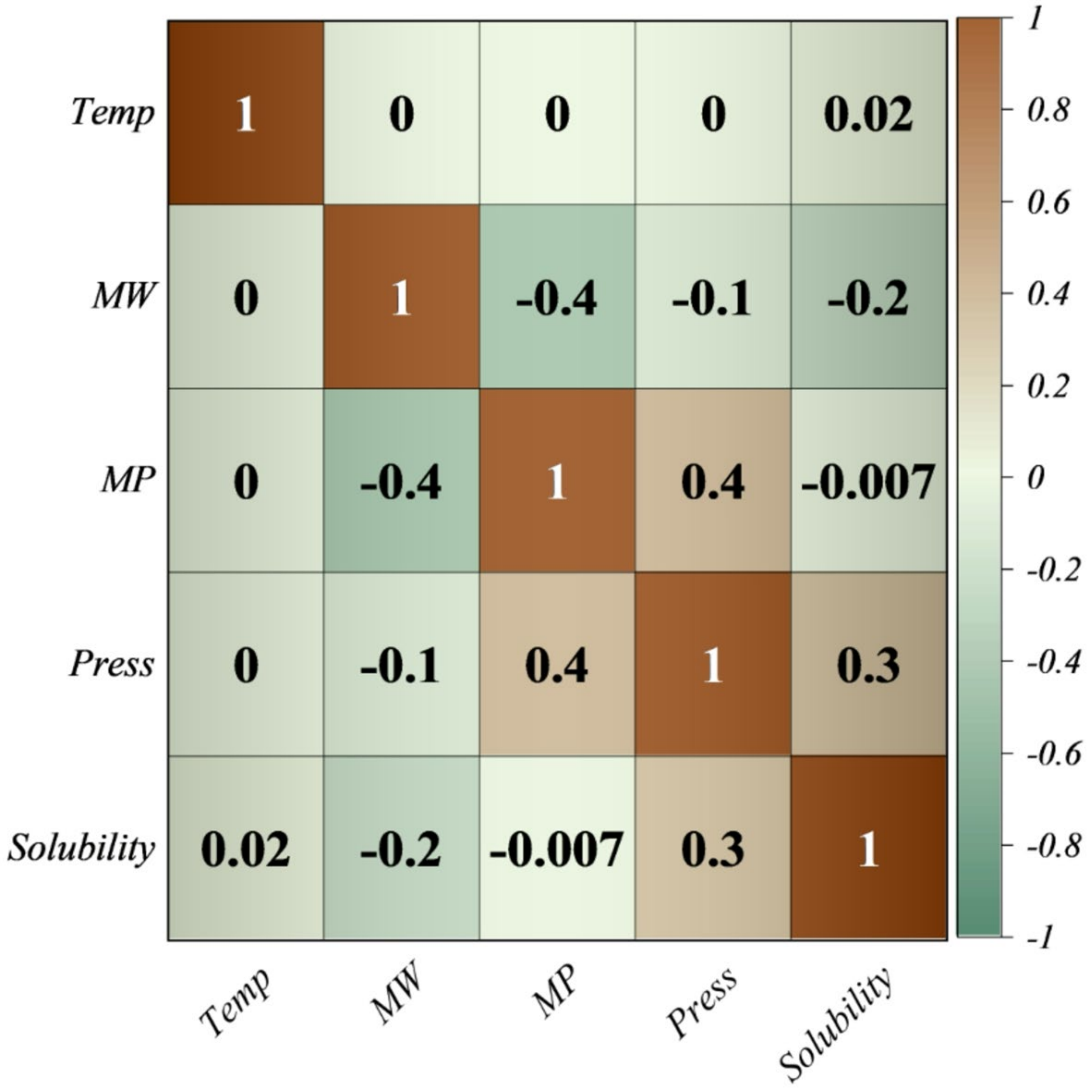
Correlation plot (Kendall) illustrating the pairwise relationships between features.

The Kendall correlation matrix (Fig. 2) reveals that solubility is most positively influenced by pressure (τ ≈ 0.3), moderately hindered by molecular weight (τ ≈ − 0.2), and only weakly affected by temperature (τ ≈ 0.02) or melting point (τ ≈ − 0.007), while inter-feature associations such as the negative correlation between molecular weight and melting point (–0.4) and the positive correlation between melting point and pressure (+ 0.4) highlight the intertwined nature of molecular and thermodynamic factors, thereby underscoring the need for multi-variable predictive modeling rather than reliance on single-parameter trends.

**Fig. 3:**
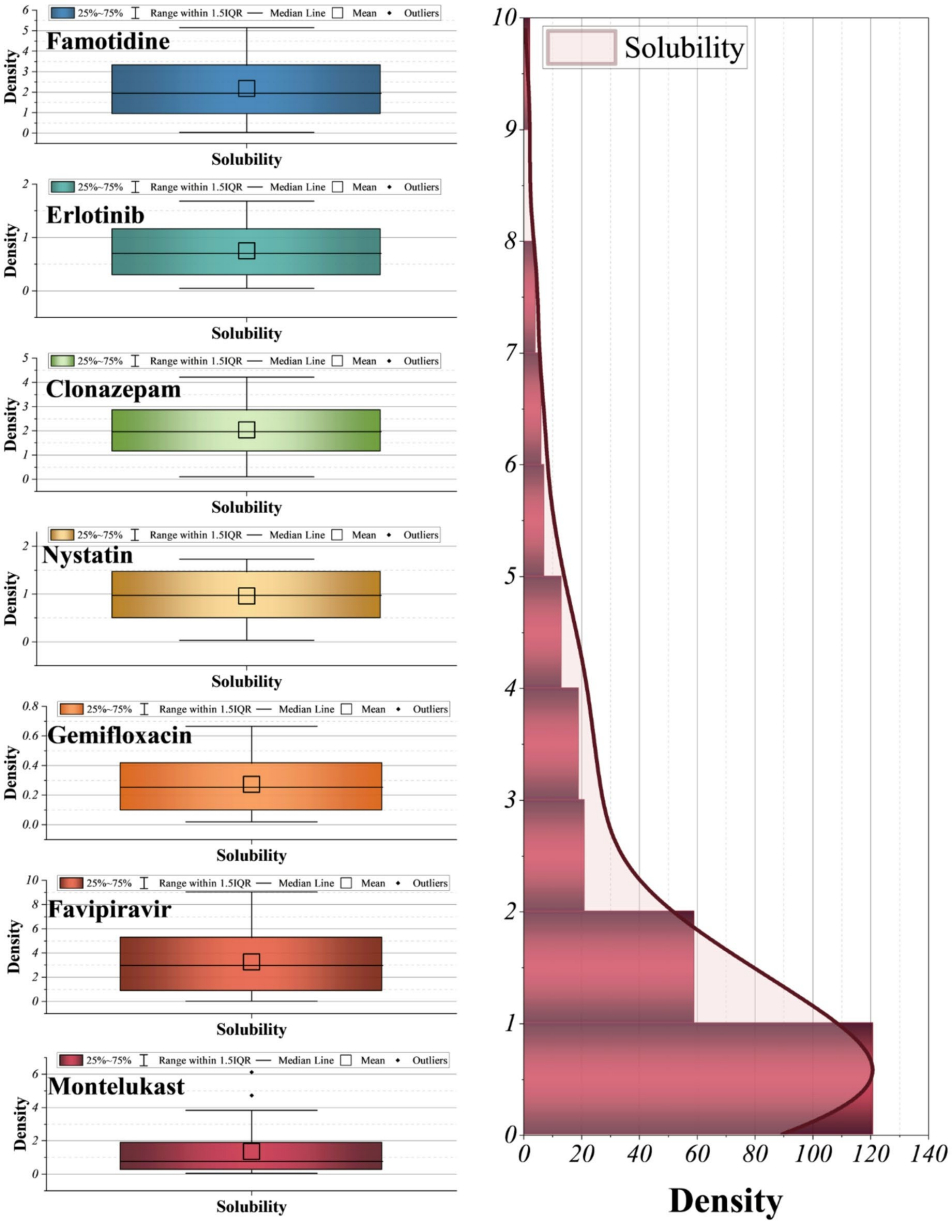
Histogram plot with kernel smoothing for the randomized target distribution, alongside box plots illustrating the target distribution across seven drugs.

The combined histogram and box plots in Fig. 3 highlight the heterogeneity of solubility values across the seven investigated drugs, where the overall distribution of solubility is highly right-skewed, with the majority of experimental values clustered in the low range (below 20 g/L × 10) and only a few extreme cases extending toward much higher solubility, reflecting a realistic but imbalanced dataset; drug-specific box plots further reveal substantial variability, with relatively narrow ranges for famotidine, clonazepam, and nystatin, broader dispersions for gemifloxacin and favipiravir, and particularly pronounced variability and outliers in montelukast, indicating that different structural and thermodynamic profiles produce distinct solubility behaviors, which in turn emphasizes the necessity of robust ML approaches capable of handling skewed distributions and drug-specific heterogeneity rather than relying on uniform statistical assumptions.

## Methodology

### Machine learning models

#### Support vector regression (SVR)

SVR is an extension of the Support Vector Machine framework for regression analysis. It assumes a functional relationship between the independent variables $\left(X\right)$and the dependent variable $\left(Y\right),$ where the objective is to find a regression function that deviates from the observed values by no more than a specified tolerance $\left(\epsilon\right).$ To achieve this, SVR employs Vapnik’s $\epsilon-$insensitive loss function, where prediction errors smaller than ε are ignored, and only deviations greater than ε contribute to the loss. The SVR optimization problem minimizes a cost function consisting of two parts: (i) a penalty proportional to the magnitude of the regression coefficients, which controls model complexity and prevents overfitting, and (ii) a penalty on deviations larger than ε, weighted by the regularization parameter $\left(C\right).$ This dual objective ensures a balance between model smoothness and predictive accuracy.

In SVR, only a subset of training points, known as *support vectors*, influence the regression function. These are the samples with prediction errors exceeding ε, and they define the optimal regression hyperplane. To capture nonlinear relationships in solubility data, kernel functions are introduced. In this study, the radial basis function kernel was adopted due to its strong performance in modeling nonlinear patterns in physicochemical datasets. The main hyperparameters $C$ (regularization strength), $\gamma$ (kernel width), and $\epsilon$ (error margin) are problem-dependent and were tuned using bio-inspired optimizers. This allowed the SVR model to adapt effectively to the complex, nonlinear relationships governing drug solubility in supercritical carbon dioxide^36^.

#### Extreme gradient boosting regression (XGBR)

Models for predicting missing laboratory test data were trained using XGBoost, a supervised learning approach. The enhanced distributed gradient boosting library XGBoost was selected because to its value in model training^37^. To create an ideal model, this method repeatedly selects the optimal split at each step using an adaptive binary splitting algorithm. XGBoost’s tree-based architecture makes it resistant to overfitting and outliers, which improves model selection processes. The normalized goal of the XGBoost model during the ${s}^{th}$ training phase is defined by Eq. (1). The difference between the predicted value ${{y}^{\left(s\right)}}_{p}$ and the associated ground truth ${y}_{m}$ is measured by the loss function $\mathcal{L}\mathcal{f}\left({{y}^{\left(s\right)}}_{p},{y}_{m}\right)$.

1 $${\mathcal{L}\mathcal{f}}^{\left(s\right)}=\sum_{i}\mathcal{l}\left({{y}^{\left(s\right)}}_{p},{y}_{m}\right)+\sum_{q}{\Omega}\left({f}_{q}\right)$$

$\left\| \omega \right\|^{2}$ is the $\mathcal{L}\mathcal{f}2$ norm of all leaf scores in training examples, where T is the number of leaves. A regularizer that captures the complexity of the ${q}^{th}$ tree is ${\Omega}\left({f}_{q}\right)=\gamma T+\frac{1}{2}\lambda \left\| \omega \right\|^{2}$. The precision of the tree search is determined by the parameters $\gamma$ and $\lambda$.

#### XGSV ensemble (non-hybrid)

The XGSV model integrates the complementary strengths of XGBR and SVR through a pure ensemble strategy, without any optimizer-driven hybridization^38–40^. In this framework, both constituent learners are trained independently on the identical feature space and target variable. Their individual predictions are then fused by a weighted averaging scheme in which the weights are optimized via internal cross-validation to minimize root-mean-square error (RMSE). This design ensures that the ensemble capitalizes on the nonlinear pattern-capturing ability and high variance-reduction of XGBR, while simultaneously preserving the margin-based generalization and smoothness control inherent to SVR. From a methodological perspective, the ensemble acts as a variance–bias compensator. XGBR, while powerful in modeling complex interactions among molecular descriptors and thermodynamic parameters, can exhibit localized overfitting in sparsely populated regions of the feature space. SVR, conversely, is more resistant to overfitting but may underrepresent subtle higher-order interactions. By combining their outputs, XGSV reduces the predictive variance of XGBR and mitigates the bias of SVR, yielding a balanced predictive surface that remains stable across heterogeneous drug classes. The non-hybrid nature of XGSV is deliberate. No external bio-inspired optimizers are invoked beyond the base hyperparameter tuning intrinsic to each algorithm, thereby limiting computational overhead and preserving model transparency. For pharmaceutical scientists, this transparency is advantageous: the separate interpretability of the SVR coefficients and the XGBR feature-importance profiles remains intact, enabling post-hoc attribution analyses such as Feature Attribution Sensitivity Test (FAST) or cosine amplitude mapping to be applied directly to each component. Consequently, XGSV provides a tractable yet robust predictive tool, striking a pragmatic balance between accuracy, interpretability, and computational efficiency qualities essential for deployment in early-stage drug formulation workflows where reproducibility and explanatory clarity are as critical as raw predictive performance.

### Optimizers for hyperparameter tuning

Hyperparameter optimization for the SVR and XGBoost models was performed using two bio-inspired algorithms, Greylag Goose Optimization (GGO) and Horned Lizard Optimization Algorithm (HLOA). For each candidate solution generated by the optimizer, a full model was trained and evaluated using five-fold cross-validation on the training subset. The fitness of each candidate was defined through a multi-objective formulation that minimized RMSE while maximizing R^2^, and Pareto dominance was used to guide the search toward non-dominated solutions. All simulations were conducted in a Python 3.10 environment using scikit-learn (v1.4) for SVR, XGBoost (v1.7) for gradient boosting, and custom Python implementations of the GGO and HLOA algorithms. Computations were carried out on a workstation equipped with an Intel Core i7-12700 H CPU (14 cores, 2.3–4.7 GHz) and 16 GB RAM under Windows 11 (64-bit). No GPU acceleration was used. Detailed optimization process are available as a supplementary material.

#### Greylag Goose Optimization (GGO)

Greylag Goose Optimization (GGO) is a population-based metaheuristic inspired by the collective foraging and movement behavior of greylag geese^41,42^. Candidate solutions represent geese, and the algorithm balances exploration (global search) and exploitation (local refinement) to avoid local optima and approach the global optimum^43^.

- *Exploration phase* Agents update their positions relative to the current best solution (leader) using random control parameters, encouraging global search. Additional diversity is introduced by combining positions of randomly selected agents and by spiral-like movements that enhance space coverage and prevent premature convergence.
- *Exploitation phase* Poorer agents (non-sentries) move toward promising regions guided by three elite agents (sentries). Their positions are updated based on distance and direction to these sentries, and the final update is obtained by averaging the three guided movements, refining solutions near the best candidates.
- *Local space exploration near the leader* Agents search around the best solution using a distance-based update with a time-varying control factor, gradually shifting from exploration to exploitation as iterations progress.

**Fig. 4:**
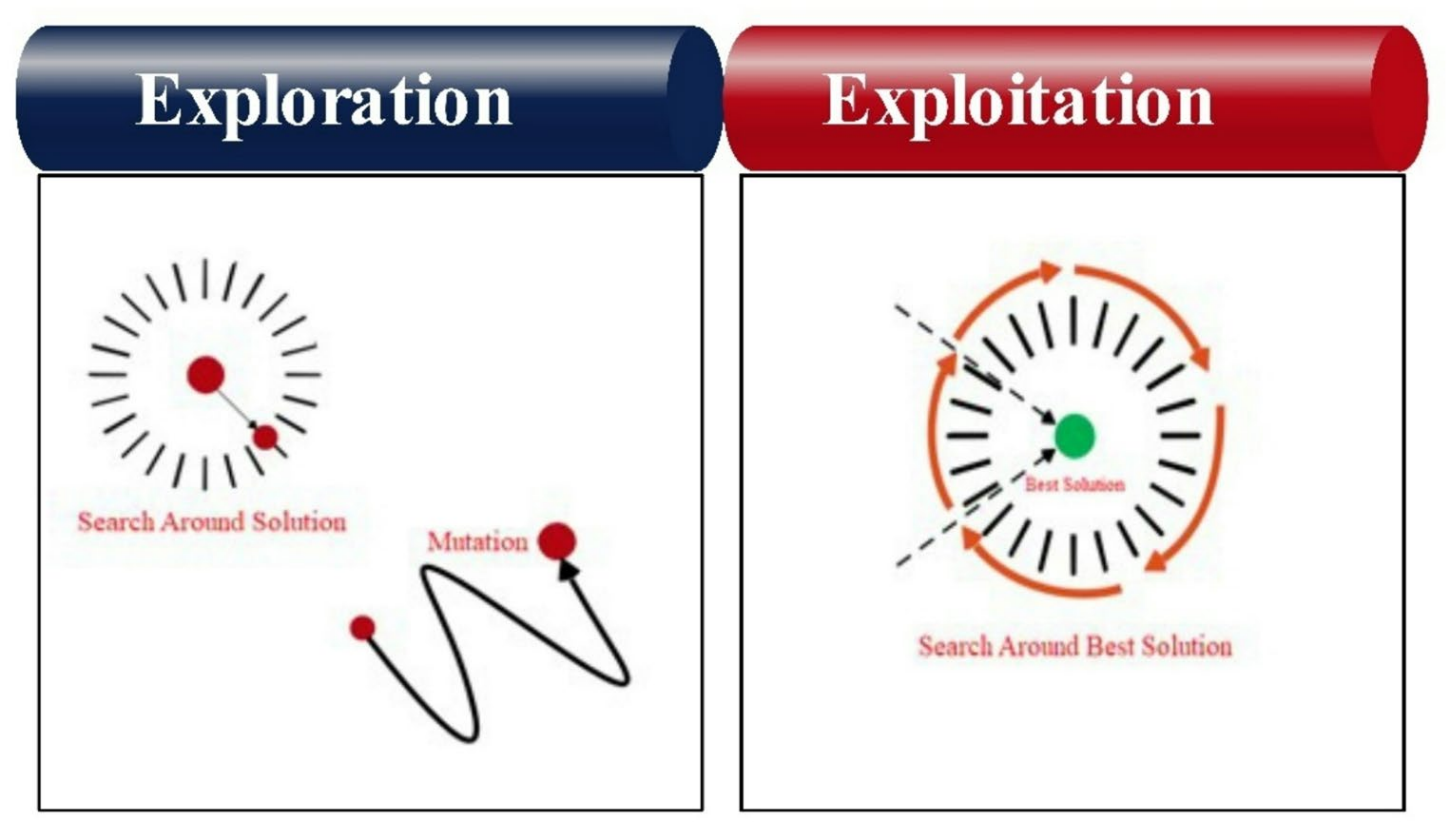
The GGO $$\mathrm{e}\mathrm{x}\mathrm{p}\mathrm{l}\mathrm{o}\mathrm{i}\mathrm{t}\mathrm{a}\mathrm{t}\mathrm{i}\mathrm{o}\mathrm{n}\mathrm{a}\mathrm{n}\mathrm{d}\mathrm{e}\mathrm{x}\mathrm{p}\mathrm{l}\mathrm{o}\mathrm{r}\mathrm{a}\mathrm{t}\mathrm{i}\mathrm{o}\mathrm{n}$$.

Overall, GGO iteratively updates solutions using leader guidance, randomization, and multi-agent cooperation to achieve an effective balance between global exploration and local exploitation. The exploration and exploitation behaviors of the GGO algorithm are examined in Fig. 4.

#### Horned Lizard Optimization Algorithm (HLOA)

The Horned Lizard Optimization Algorithm (HLOA)^44^ is inspired by the horned lizard’s survival mechanisms camouflage, skin color adaptation, blood-squirting defense, and evasive movement and translates them into mathematical update rules for optimization.

- *Crypsis strategy* Models camouflage using sinusoidal and cosine-based position updates guided by the current best solution and randomly selected agents to enhance exploration.
- *Skin lightening/darkening* Mimics thermoregulation by generating new candidate solutions around the best agent and replacing the worst agent, using random “light” or “dark” coefficients.
- *Blood-squirting strategy* Uses projectile-motion equations to update agent positions, balancing attraction to the best solution and retention of current positions.
- *Move-to-escape (M-to-E)* Combines local random walks with global guidance toward the best solution.
- *σ-melanophore hormone strategy* Normalizes fitness to control exploration; poorly performing agents are re-positioned using differences between random agents when exploration drops below a threshold.

Overall, HLOA iteratively applies these biologically inspired strategies, selects updates probabilistically, enforces boundary constraints, and replaces poor solutions to achieve a balance between exploration and exploitation (Algorithm 1).

**Algorithm 1:**
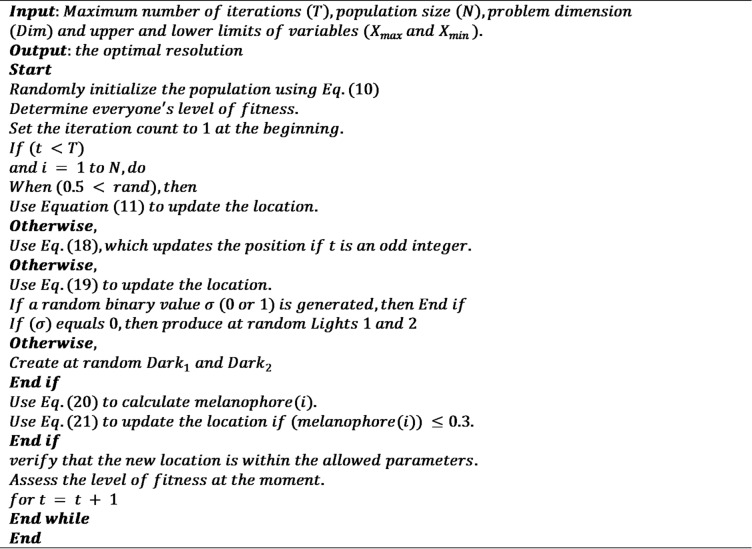
The HLOA pseudo code.

#### Selection of bio-inspired optimizers

The GGO and HLOA were chosen for hyperparameter tuning due to their superior balance between global exploration and local exploitation, which is critical for high-dimensional, nonlinear search spaces typical in ensemble machine learning models. Compared with classical optimizers such as Particle Swarm Optimization (PSO) or Genetic Algorithms (GA), GGO and HLOA have demonstrated faster convergence and higher robustness against premature convergence in complex optimization landscapes. Specifically, GGO mimics the migratory and foraging strategies of greylag geese, efficiently navigating large search spaces while avoiding local optima. HLOA leverages behavioral and physiological traits of horned lizards to adaptively adjust search dynamics, enabling fine-tuning of parameters for enhanced predictive performance. Preliminary benchmarking on the solubility dataset confirmed that both optimizers achieved lower validation errors and more consistent results than PSO and GA under identical conditions. Therefore, integrating GGO and HLOA into the proposed framework ensures reliable hyperparameter optimization, improving model accuracy and generalization while maintaining computational efficiency.

### Multi-objective optimization

Predicting solubility in supercritical carbon dioxide involves competing objectives: the models must minimize predictive error while simultaneously maximizing explanatory power. These goals, quantified respectively by the coefficient of determination (R^2^) and root-mean-square error (RMSE), cannot generally be optimized in isolation, because improving one metric often degrades the other. To address this challenge, model calibration was formulated as a multi-objective optimization (MOO) problem and resolved through Pareto-dominance analysis.

In this framework, every candidate solution defined by a specific set of hyperparameters for SVR, XGBR, or their optimized hybrids corresponds to a point in the two-dimensional objective space (RMSE, R^2^). A solution A is said to dominate solution B when it achieves equal or lower RMSE and equal or higher R^2^, with at least one strict improvement. The Pareto front is constructed as the set of non-dominated solutions representing the best achievable tradeoffs between accuracy and generalization. Hyperparameter search procedures for the GGO and HLOA were guided not by a single scalar objective, but by this dominance criterion.

The Pareto-based strategy offers several advantages for pharmaceutical modeling. First, it avoids the need to impose arbitrary weights on RMSE and R^2^, allowing the data itself to reveal the feasible accuracy–interpretability frontier. Second, it provides a portfolio of optimal solutions rather than a single point estimate, enabling the selection of models that best match specific experimental priorities, for example, choosing a slightly higher RMSE if a larger R^2^ and stronger mechanistic insight are required for regulatory justification. Finally, visualizing the Pareto front exposes regions of diminishing returns, clarifying where additional computational effort produces negligible gains in prediction quality.

By embedding this MOO approach into the training pipeline, the final SVR, XGBR, and hybrid models reflect a balanced compromise between predictive precision and explanatory robustness, a prerequisite for reliable deployment in drug-formulation research where both accuracy and scientific interpretability are paramount.

### Evaluation metrics

Model performance for solubility prediction was assessed using a comprehensive suite of statistical indicators to capture both absolute error and relative explanatory power. The selected metrics, $RMSE,$ ratio of $RMSE$ to the standard deviation of observations $\left(RSR\right)$, R^2^, mean absolute relative error $\left(MARE\right),$mean absolute error $\left(MAE\right),$ and Theil’s inequality coefficient $\left(TIC\right),$ provide complementary perspectives on accuracy, precision, and predictive reliability.

RMSE:

Quantifies the square root of the average squared deviation between predicted $\left({y}^{i}\right)$ and experimental $\left({y}^{i}\right)$ solubility values. Lower $RMSE$indicates smaller residual variance and higher fidelity across operating conditions.

R^2^:

Measures the proportion of total variance in the experimental dataset explained by the model. Values approaching 1.0 signify strong explanatory capacity and effective representation of nonlinear relationships.

MAE:

Provides the mean of absolute prediction errors, emphasizing overall accuracy without disproportionately weighting large deviations.

RSR:

Normalizes RMSE by the standard deviation of the measured solubility values $\left({s}_{y}\right)$, enabling direct comparison across datasets of different scales.

MARE:

Expresses the average absolute error as a fraction of the observed values, providing a dimensionless measure of relative predictive performance that is important for process design.

Theil’s Inequality Coefficient (TIC):

Evaluates overall forecasting quality by comparing the magnitude of prediction errors to the combined variability of observed and predicted data. A TIC approaching zero indicates near-perfect agreement.

For a dataset of $n$ observations with experimental values ${y}_{i}$​, predictions $\widehat{{y}_{i}}$, and mean of observed values $\stackrel{-}{y}$. Table 2 shows the statistical performance indicators^45^.

**Table 2:** Statistical performance indicators used for model evaluation.

Equation	Definition / Description	Acceptance Threshold
$RMSE=\sqrt{\frac{1}{n}{\sum}_{i=1}^{n}{({y}_{i}-\widehat{{y}_{i}})}^{2}}$	Measures the average magnitude of prediction error; sensitive to large deviations.	Lower is better; typically, RMSE → 0 desirable
${R}^{2}=1-\frac{{\sum}_{i=1}^{n}{({y}_{i}-\widehat{{y}_{i}})}^{2}}{{\sum}_{i=1}^{n}{({y}_{i}-\stackrel{-}{y})}^{2}}$	Proportion of variance explained by the model.	R^2^ ≥ 0.80 (good), ≥ 0.90 (excellent)
$MAE=\frac{1}{n}{\sum}_{i=1}^{n}|{y}_{i}-\widehat{{y}_{i}}|$	Average absolute deviation between predicted and observed values	Average magnitude of absolute errors; easy to interpret.
$RSR=\frac{RMSE}{{s}_{y}},{s}_{y}=\sqrt{\frac{1}{n-1}\sum_{i=1}^{n}{({y}_{i}-\stackrel{-}{y})}^{2}}$	Normalizes RMSE; enables cross-dataset comparison.	Percent-based error measure relative to observed values.
$MARE=\frac{1}{n}\sum_{i=1}^{n}\left|\frac{{y}_{i}-\widehat{{y}_{i}}}{{y}_{i}}\right|$	Same as above, expressed as a percentage.	RSR ≤ 0.70 (acceptable); ≤ 0.50 good
$TIC=\frac{\sqrt{\frac{1}{n}\sum_{i=1}^{n}{({y}_{i}-\widehat{{y}_{i}})}^{2}}}{\sqrt{\frac{1}{n}\sum_{i=1}^{n}{y}_{i}^{2}}+\sqrt{\frac{1}{n}\sum_{i=1}^{n}{\widehat{{y}_{i}}}_{i}^{2}}}$	Relative metric assessing forecasting quality.	TIC < 0.3 acceptable; → 0 ideal

These expressions ensure consistent, quantitative comparison of model accuracy, stability, and predictive robustness across all experimental and validation phases.

## Results and discussion

### Model benchmarking (baseline performance)

**Fig. 5:**
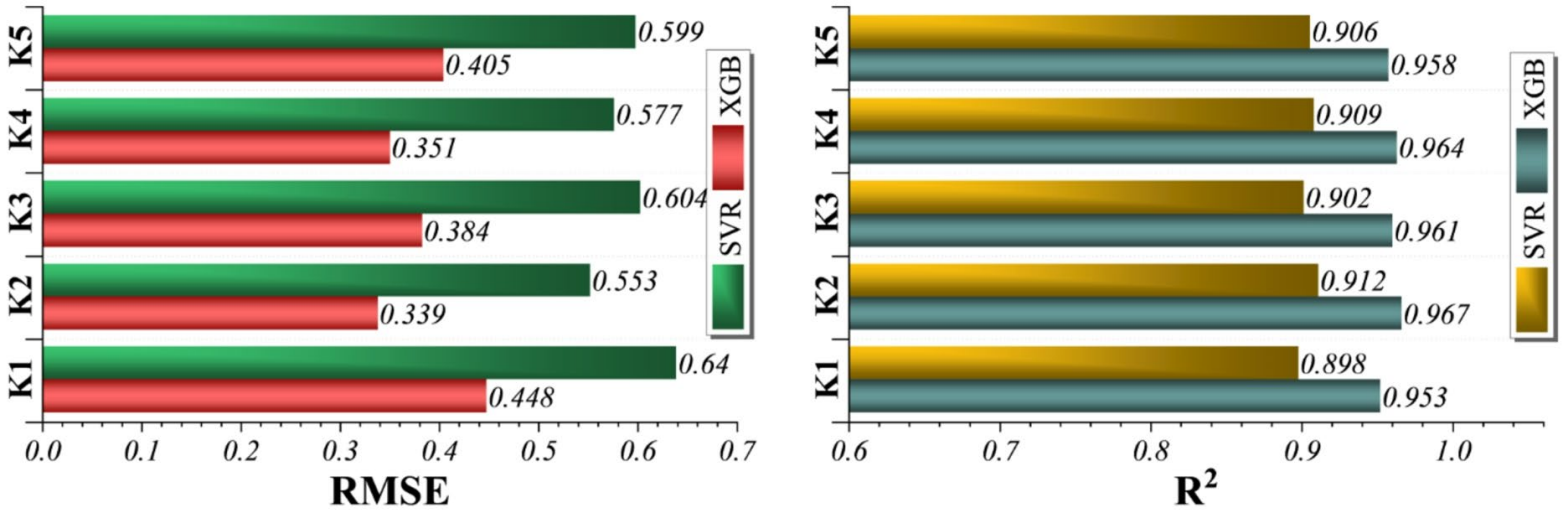
Bar plot for k-fold cross-validation performance across models.

Figure 5 presents the results of k-fold cross-validation, comparing the predictive performance of SVR and XGB models using RMSE and R^2^ metrics. The compiled dataset contained *N* = 252 experimental solubility measurements obtained from six independent published studies. To ensure rigorous and unbiased evaluation, the data were divided into training (80%), validation (10%), and test (10%) subsets using stratified random sampling based on pressure and solubility distributions. This resulted in 202 samples for training, 25 samples for validation, and 25 samples for final testing. In addition to the fixed split, model robustness was assessed using 5-fold cross-validation (CV) applied only to the training portion. In each fold, approximately 161 samples were used for training and 41 samples for internal validation, while the external test set remained untouched throughout optimization. The GGO and HLOA optimizers relied solely on the validation subset within each CV fold to guide hyperparameter tuning and prevent data leakage.

**Fig. 6:**
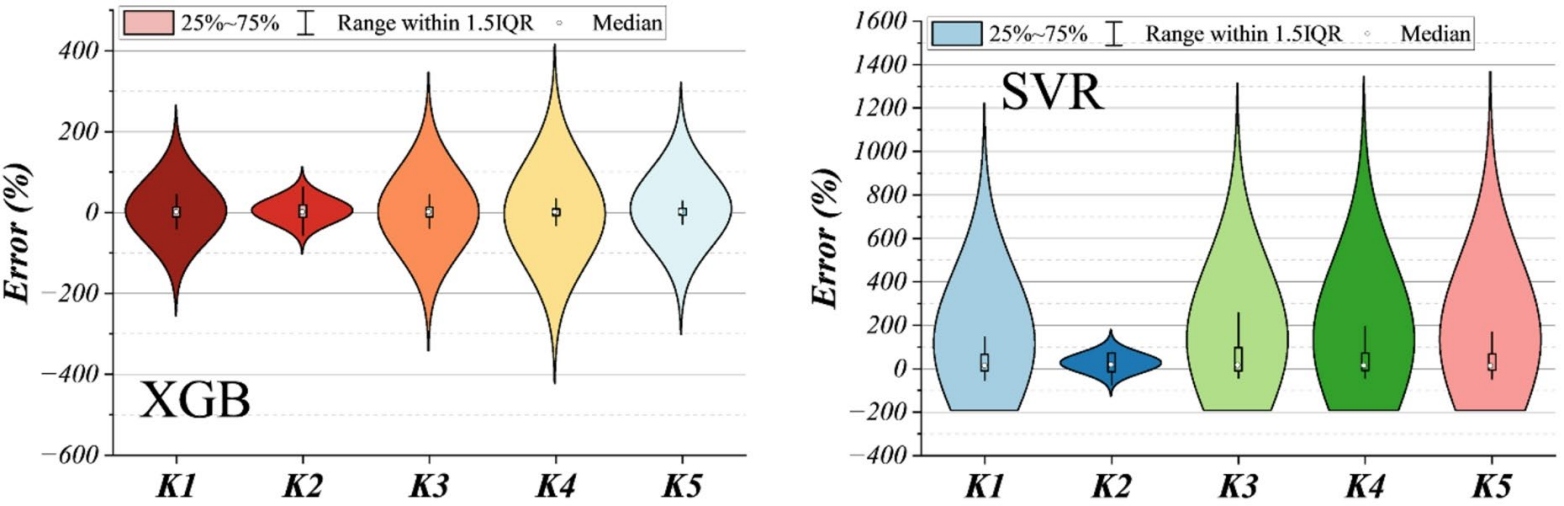
Obtained error percentage in all 5 folds.

Across all five folds, XGB consistently outperforms SVR, achieving lower RMSE values (0.339–0.604 vs. 0.351–0.64) and higher coefficients of determination (0.953–0.967 vs. 0.898–0.964). The relatively stable performance across folds indicates robust generalization of both models, but the sharper reduction in error and higher explanatory power of XGB highlights its superior capability to capture the complex, nonlinear dependencies between molecular and thermodynamic features. These results confirm that ensemble boosting strategies are better suited than kernel-based methods for solubility prediction in diverse drug–condition datasets. In addition, Fig. 6 shows the obtained error percentage in all 5 folds.

Table 3 summarizes the predictive performance of single, hybrid, and ensemble models across training, validation, and test phases using six statistical indicators. Single models provided a useful benchmark. XGB consistently outperformed SVR, with lower RMSE and higher R^2^ across all stages (e.g., test R^2^ of 0.927 vs. 0.916). This reflects XGB’s ability to capture nonlinear thermodynamic–molecular interactions more effectively, such as the nonlinear influence of temperature and pressure on solubility. SVR, while stable, struggled with error magnitude (test MAE = 0.307), suggesting limited flexibility when extrapolating across diverse drug classes. Hybrid models showed a clear improvement, particularly XGB+HLOA (XGHL) and XGB + GOA (XGGO), which combined ML with nature-inspired optimization. XGHL achieved the best overall performance (test RMSE = 0.210, R^2^ = 0.975), indicating that hyperparameter tuning via HLOA enabled the model to generalize solubility trends more accurately. This accuracy is significant from a pharmacological perspective: an RMSE reduction of ~ 40% compared to baseline XGB means fewer false predictions of poorly soluble compounds, which can reduce costly wet-lab screening failures. XGGO also delivered strong results (test R^2^ = 0.967), highlighting the robustness of optimizer-guided learning.

The SVR-based hybrids (SVR+HLOA (SVHL) and SVR + GOA (SVGO)) improved over plain SVR but remained weaker than their XGB counterparts. This suggests that while optimization enhanced parameter selection, SVR’s kernel-based approach was less adaptable to the multi-dimensional relationships between molecular weight, melting point, and solubility. The ensemble model (XGB + SVR (XGSV)) performed moderately well, averaging the strengths of XGB and SVR (test R^2^ = 0.934). However, it did not surpass the optimized hybrids, likely because error reduction in SVR limited the ensemble’s gains. From an industrial standpoint, this indicates that ensembles may not always be the best strategy if one constituent model is consistently weaker.

Although the hybrid XGBoost-based models yield only moderate numerical improvements in conventional performance metrics relative to baseline models, the added modeling complexity is justified by gains in robustness and stability. The bio-inspired optimization algorithms systematically explore the hyperparameter space and reduce dependence on manual tuning or arbitrary parameter selection. In addition, the ensemble structure mitigates overfitting and decreases prediction variance across different data partitions. These advantages are particularly important in the present context, where the dataset is relatively small and unevenly distributed across compounds. Consequently, the proposed hybrid framework offers a more reliable and reproducible modeling strategy rather than merely incremental error reduction.

To ensure that the performance gains obtained using GGO and HLOA are not simply a consequence of applying any metaheuristic optimizer, two classical and widely adopted optimization algorithms, Genetic Algorithm (GA) and Particle Swarm Optimization (PSO), were additionally implemented for hyperparameter tuning of the XGB and SVR models. This extended benchmarking enables direct comparison between recent bio-inspired optimizers and conventional methods under identical data partitions and evaluation metrics. The results indicate that GA and PSO yield noticeable improvements over unoptimized models; however, GGO- and HLOA-optimized models consistently achieve lower RMSE and higher R^2^ across all datasets. This demonstrates that the marginal gains observed are systematic rather than incidental and that the newer optimizers provide a more effective exploration–exploitation balance for the present solubility prediction task.

**Table 3:** Performance metrics of baseline, classical-optimizer-based (GA, PSO), and bio-inspired-optimizer-based (GGO, HLOA) hybrid models.

Process	Category	Models	Evaluation metrics					
			RMSE	R^2^	MAE	RSR	MARE	TIC
Train	Single Models	XGB	0.346	0.969	0.229	0.177	0.240	0.066
		SVR	0.576	0.914	0.429	0.295	0.429	0.110
	Hybrid Models	XGHL	0.149	0.995	0.090	0.077	0.104	0.028
		XGGO	0.252	0.984	0.166	0.129	0.170	0.048
		SVHL	0.433	0.952	0.274	0.222	0.361	0.081
		SVGO	0.482	0.940	0.316	0.247	0.320	0.092
	Classical-Optimizers	XGGA	0.281	0.981	0.188	0.144	0.195	0.052
		XGPSO	0.265	0.983	0.176	0.136	0.183	0.049
	Ensemble Model XGB + SVR	XGSV	0.370	0.965	0.263	0.190	0.241	0.071
Validation	Single Models	XGB	0.255	0.972	0.173	0.191	0.283	0.066
		SVR	0.499	0.868	0.361	0.375	0.486	0.132
	Hybrid Models	XGHL	0.172	0.984	0.117	0.129	0.225	0.045
		XGGO	0.252	0.966	0.183	0.189	0.273	0.066
		SVHL	0.294	0.952	0.199	0.221	0.290	0.078
		SVGO	0.395	0.913	0.244	0.297	0.355	0.104
	Classical-Optimizers	XGGA	0.225	0.975	0.153	0.170	0.255	0.058
		XGPSO	0.213	0.978	0.145	0.160	0.242	0.055
	Ensemble Model XGB + SVR	XGSV	0.328	0.943	0.232	0.246	0.301	0.086
Test	Single Models	XGB	0.357	0.927	0.235	0.270	0.282	0.099
		SVR	0.397	0.916	0.307	0.300	0.422	0.107
	Hybrid Models	XGHL	0.210	0.975	0.120	0.158	0.173	0.058
		XGGO	0.244	0.967	0.160	0.184	0.228	0.067
		SVHL	0.403	0.913	0.236	0.305	0.266	0.110
		SVGO	0.384	0.924	0.279	0.290	0.394	0.109
	Classical-Optimizers	XGGA	0.263	0.961	0.173	0.199	0.245	0.071
		XGPSO	0.251	0.964	0.165	0.190	0.236	0.069
	Ensemble Model XGB + SVR	XGSV	0.342	0.934	0.258	0.258	0.307	0.094

**Fig. 7:**
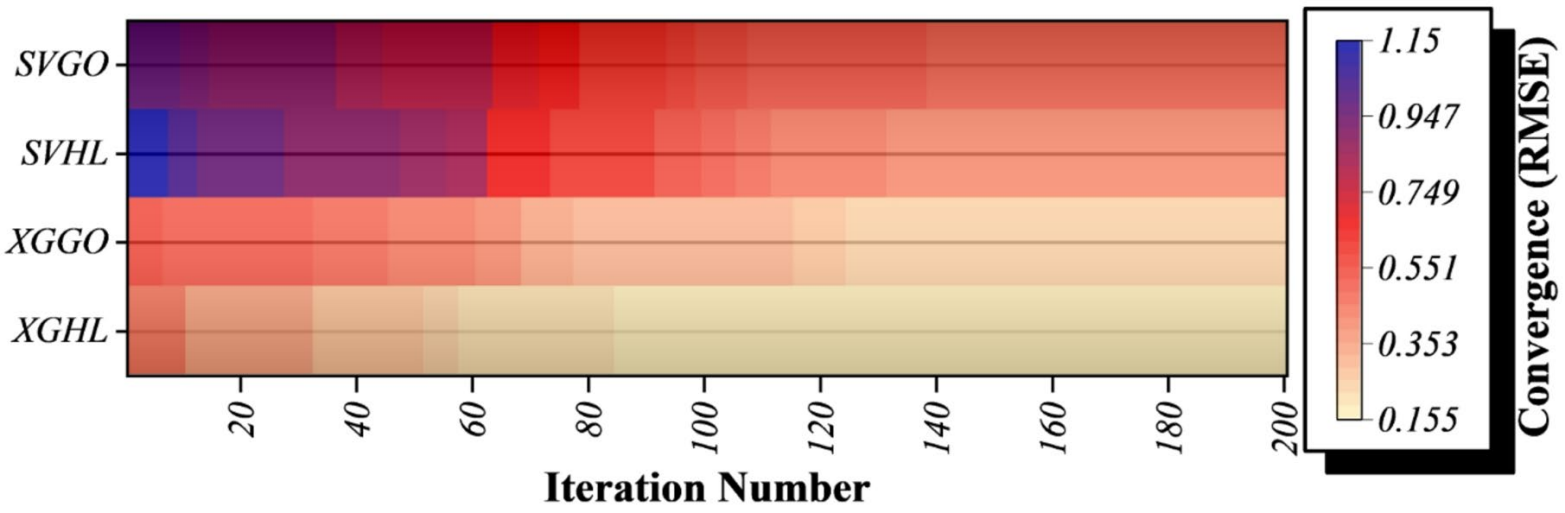
Heatmap for the convergence behavior of the models during optimization.

**Fig. 8:**
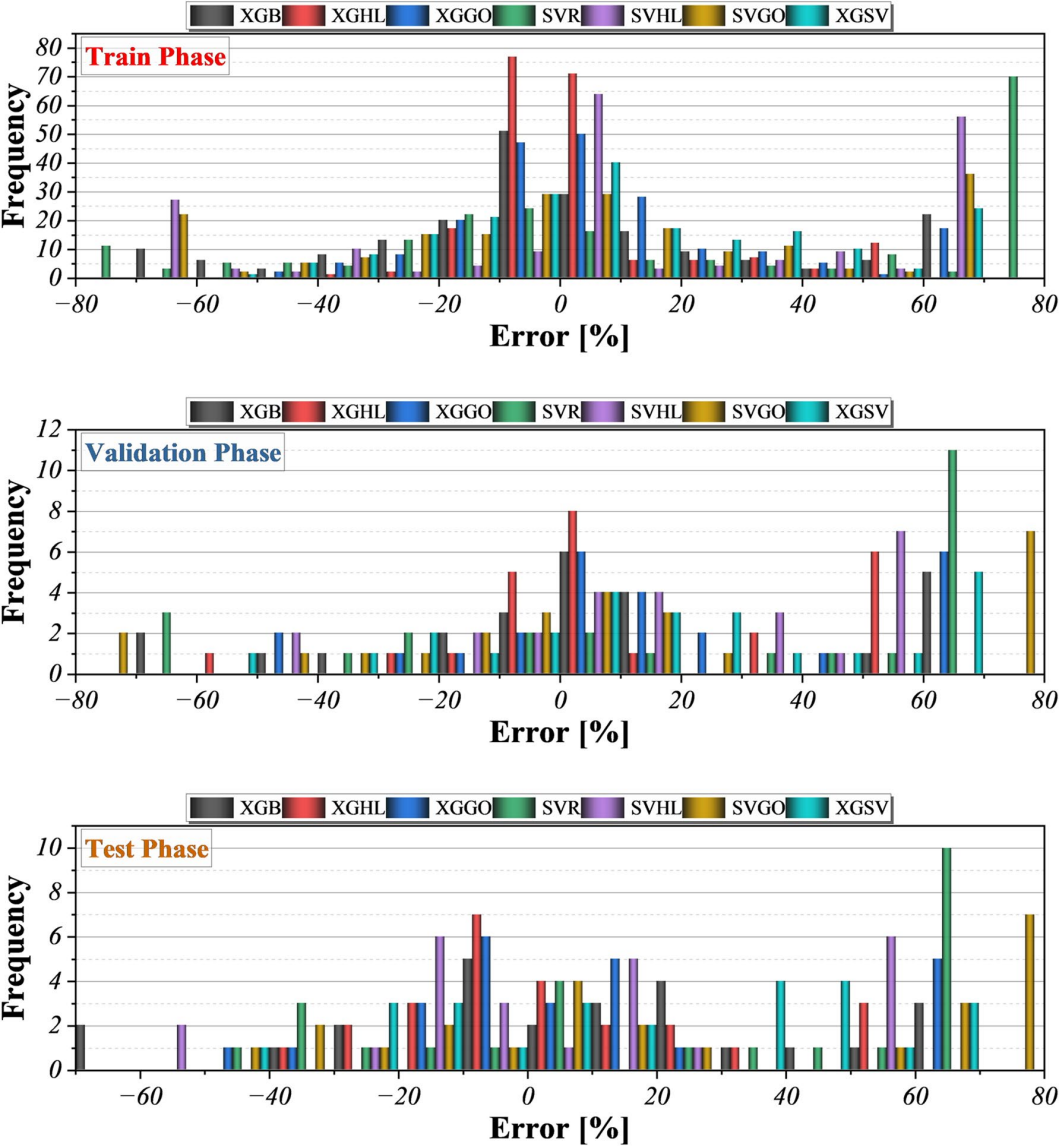
Histogram plot for the frequency distribution of prediction errors.

**Fig. 9:**
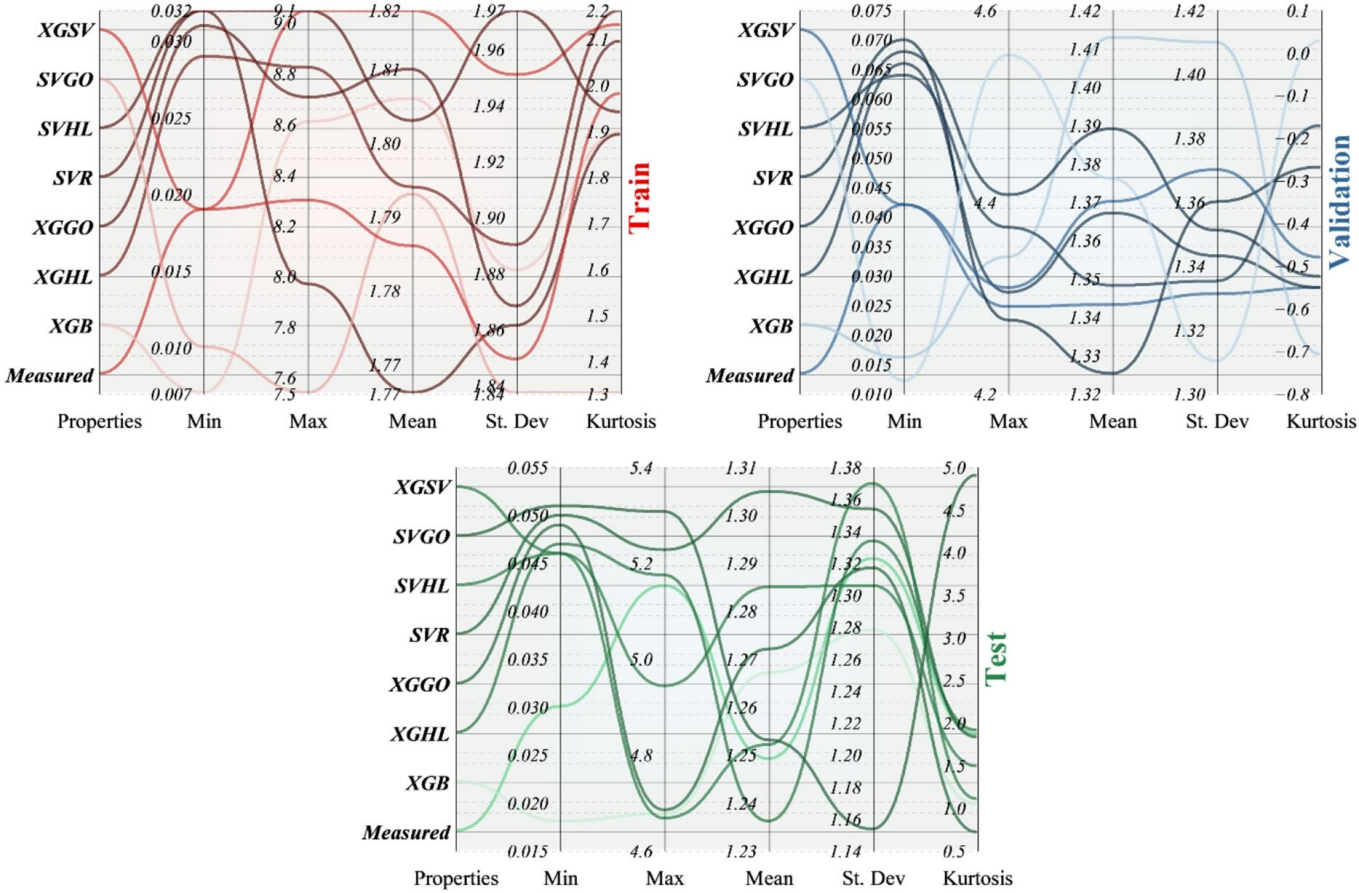
Histogram plot for the frequency distribution of prediction errors.

The convergence behavior of all hybrid models during the 200 optimization iterations is illustrated in Fig. 7. These plots show the evolution of the objective function (RMSE) over successive iterations, allowing assessment of both convergence speed and stability. Error frequency distributions and learning behavior across the training, validation, and test phases are further presented in Figs. 8 and 9, providing visual evidence of model robustness and generalization. The color gradient encodes the instantaneous RMSE, with deep blue tones indicating higher residual variance and warm beige tones reflecting progressive error minimization. A pronounced stratification emerges: the XGBoost-centered hybrids (XGHL and XGGO) migrate rapidly toward the low-RMSE zone within the first 60–80 iterations, demonstrating efficient balance between exploration and exploitation by their bio-inspired optimizers. In contrast, the SVR-based hybrids (SVHL and SVGO) display a more protracted descent, suggesting slower adaptation of kernel parameters and a greater susceptibility to local optima. This differential convergence behavior highlights the structural advantages of tree-boosting models when navigating the multi-objective search landscape defined by simultaneous RMSE minimization and R^2^ maximization.

The hyperparameter search ranges used during optimization are summarized in Table 4, and the final selected values correspond to Pareto-optimal solutions identified by the multi-objective optimization framework. The XGHL model exhibits an aggressively deep ensemble (154 maximum tree depth) coupled with a high learning rate (0.933) and moderate subsampling (0.59), a combination that accelerates gradient boosting while maintaining regularization through balanced column sampling and carefully tuned ${\mathcal{l}}_{1}$​ (α = 0.001) and ${\mathcal{l}}_{2}$​ (λ = 0.856) penalties. XGGO adopts a contrasting strategy: a shallower architecture (maximum depth = 191 with fewer estimators) and a markedly lower learning rate (0.211) paired with stringent column and row subsampling, favoring conservative exploration and stronger generalization. The SVR-based hybrids converge to distinctly different regimes. SVHL requires a relatively broad ε-insensitive margin ($\epsilon$ = 3.31123) and a moderate penalty parameter ($C$= 0.1198), yielding a smoother regression surface well suited for capturing global solubility trends. SVGO, by contrast, selects a tighter error margin ($\epsilon$= 0.5500) and a slightly lower C (0.0971) but a very high kernel scale ($\gamma$ ≈ 490.7), emphasizing localized nonlinear interactions between thermodynamic descriptors and molecular features.

Taken together, the heat-map dynamics and the optimized hyperparameters reveal a feature-driven hierarchy: XGHL achieves the most decisive and stable convergence, XGGO balances accuracy with generalization, while SVR hybrids trade rapid convergence for smoother, more globally oriented fits. These characteristics explain the superior predictive performance of the XGBoost-driven hybrids in subsequent solubility forecasting, where capturing complex, high-dimensional feature relationships is critical for pharmaceutical process design.

**Table 4:** Hyperparameters of the hybrid models with their assigned values.

Hybrid Models	Hyperparameter						
	*N* estimators	Max depth	Learning rate	Colsample bytree	Subsample	Reg alpha	Reg lambda
XGHL	719	154	0.933	0.447	0.59	0.001	0.856
XGGO	419	191	0.211	0.087	0.074	0.047	0.072
Search Range Used During Optimization	50–2000	2–300	0.001–1	0.1–1	0.1–1	0–1	0–10

	C		Epsilon			Gama	
SVHL	0.119783		3.31123			677.75	
SVGO	0.097054		0.550013			490.661	
Search Range Used During Optimization	0.01–1000		0.0001–10			1e-6–1000	

The integrated analysis of prediction errors and descriptive statistics offers a detailed view of how each learning strategy reproduces the experimental solubility landscape across training, validation, and testing phases.

The multi-panel histogram in Fig. 8 depicts the frequency distribution of percentage errors for all models. During the training phase, the XGBoost-based hybrids (XGHL and XGGO) generate narrow, centrally clustered error profiles, with the majority of residuals concentrated within ± 10%, indicating strong fidelity to the experimental data. SVR and its hybrids display broader, slightly skewed distributions, reflecting higher sensitivity to outliers and local fluctuations. This pattern persists, though more subtly, in the validation phase, where XGHL maintains the tightest spread and the lowest frequency of extreme deviations. In the independent test phase, error distributions widen for all models, but the XGBoost-centered hybrids continue to exhibit a pronounced peak near zero error, demonstrating superior generalization and robustness against unseen molecular–thermodynamic combinations. The ensemble model (XGSV) shows an intermediate pattern, benefiting from variance reduction but inheriting some of the wider spread of its SVR component.

Complementing these visual trends, Table 5 quantifies the statistical properties of measured and predicted solubilities. Across all phases, predicted minima and maxima remain close to experimental bounds, confirming the absence of unrealistic extrapolations. Mean values for the top-performing hybrids deviate by less than $0.1gL^{-1} \times10$ from the measured mean, underscoring unbiased central tendency. Standard deviations for XGHL and XGGO track closely with those of the observed data, demonstrating effective capture of variability without artificial smoothing. Kurtosis values further highlight distributional fidelity: in the training set, XGHL exhibits a kurtosis (≈ 2.16) nearly identical to the measured benchmark (2.13), reflecting accurate modeling of tail behavior, whereas SVGO shows a markedly lower kurtosis (≈ 1.33), indicating underestimation of extreme solubility events. Similar relationships hold in the validation and test phases, where XGHL consistently aligns most closely with experimental kurtosis and variance.

The error histograms and descriptive statistics reveal a coherent hierarchy of predictive quality. XGHL achieves the most balanced combination of low bias, controlled variance, and faithful reproduction of distributional tails, followed closely by XGGO. SVR-based hybrids exhibit wider error spreads and occasionally underestimates high-solubility outliers, while the pure ensemble XGSV provides a moderate compromise. These findings confirm that the bio-optimized XGBoost hybrids not only minimize pointwise prediction errors but also preserve the full statistical character of experimental solubility, a critical requirement for reliable application in pharmaceutical process design and risk-sensitive formulation planning.

Moreover, Fig. 9 visualizes Table 5 for better comparison of the performance of the developed models with the measured values in the three presented phases.

**Table 5:** Statistical metrics used to compare models.

Phase		Models	Properties				
			Min	Max	Mean	St. Dev	Kurtosis
Train	Measured		0.019	9.050	1.818	1.951	2.132
	Predicted	XGB	0.007	8.610	1.806	1.881	1.896
		XGHL	0.029	8.826	1.794	1.890	2.163
		XGGO	0.031	8.707	1.810	1.868	2.096
		SVR	0.032	7.966	1.766	1.861	1.893
		SVHL	0.032	9.050	1.803	1.974	1.942
		SVGO	0.010	7.536	1.793	1.837	1.329
		XGSV	0.019	8.298	1.786	1.849	1.982
Validation	Measured		0.042	4.290	1.343	1.331	-0.553
	Predicted	XGB	0.016	4.342	1.413	1.410	-0.709
		XGHL	0.066	4.305	1.367	1.343	-0.553
		XGGO	0.068	4.407	1.389	1.351	-0.526
		SVR	0.070	4.276	1.325	1.360	-0.269
		SVHL	0.064	4.373	1.348	1.335	-0.171
		SVGO	0.012	4.553	1.376	1.310	0.029
		XGSV	0.042	4.310	1.370	1.370	-0.481
Test	Measured		0.030	5.153	1.249	1.323	1.866
	Predicted	XGB	0.018	4.675	1.267	1.278	1.036
		XGHL	0.047	5.175	1.236	1.334	1.907
		XGGO	0.049	4.683	1.272	1.317	0.710
		SVR	0.050	5.228	1.305	1.354	1.827
		SVHL	0.046	4.666	1.252	1.370	1.101
		SVGO	0.051	5.308	1.253	1.153	4.909
		***XGSV***	0.046	4.943	1.285	1.306	1.486

The pairwise Wilcoxon signed-rank tests summarized in Table 6 were conducted to assess whether the observed differences in predictive performance among the optimized models were statistically meaningful. The only comparison yielding a statistically significant result was between XGHL and XGGO (*p* = 0.0022), indicating that the HLOA optimizer provides a materially superior hyperparameter configuration for the XGBoost regressor compared with GGO. This finding statistically supports the numerical performance hierarchy observed in the RMSE and R^2^ results and highlights HLOA’s stronger global search capability when tuning complex tree-based models. In contrast, all comparisons involving the SVR-based hybrids exhibited non-significant p-values (*p* > 0.18), suggesting substantial overlap in their error distributions and confirming that SVR is comparatively insensitive to the choice of optimizer. Similarly, the comparison between XGHL and the two SVR-based models was not statistically significant, despite XGHL achieving higher average accuracy, indicating that the performance advantage of XGHL over SVHL and SVGO is not strong enough to yield a statistically distinct distribution of errors. The high p-values in the XGGO–SVHL and XGGO–SVGO comparisons (0.9381 and 0.5514, respectively) further reinforce that the GGO-optimized XGB model behaves similarly to the SVR variants in terms of prediction variability. Collectively, these statistical results demonstrate that the optimization strategy exerts its greatest influence on XGBoost, where HLOA significantly improves model stability and accuracy, whereas SVR models show broadly comparable performance regardless of the optimizer used.

**Table 6:** Statistical analyses based on the Wilcoxon method.

Comparison	Statistic	*P*-Value
XGHL vs. XGGO	12,397	0.0022
XGHL vs. SVHL	13,798	0.2126
XGHL vs. SVGO	14,401	0.1842
XGGO vs. SVHL	15,849	0.9381
XGGO vs. SVGO	15,249	0.5514
SVHL vs. SVGO	14,943	0.3898

### Multi-objective optimization & Pareto analysis

Hyperparameter tuning for solubility prediction was formulated as a multi-objective optimization (MOO) problem, in which the competing goals of maximizing predictive accuracy (high R^2^) and minimizing residual error (low RMSE) were optimized simultaneously. Unlike single-objective searches that collapse these criteria into a weighted scalar, the MOO framework identifies a Pareto set of non-dominated solutions, each indicating an optimal tradeoff between accuracy and generalization.

**Fig. 10:**
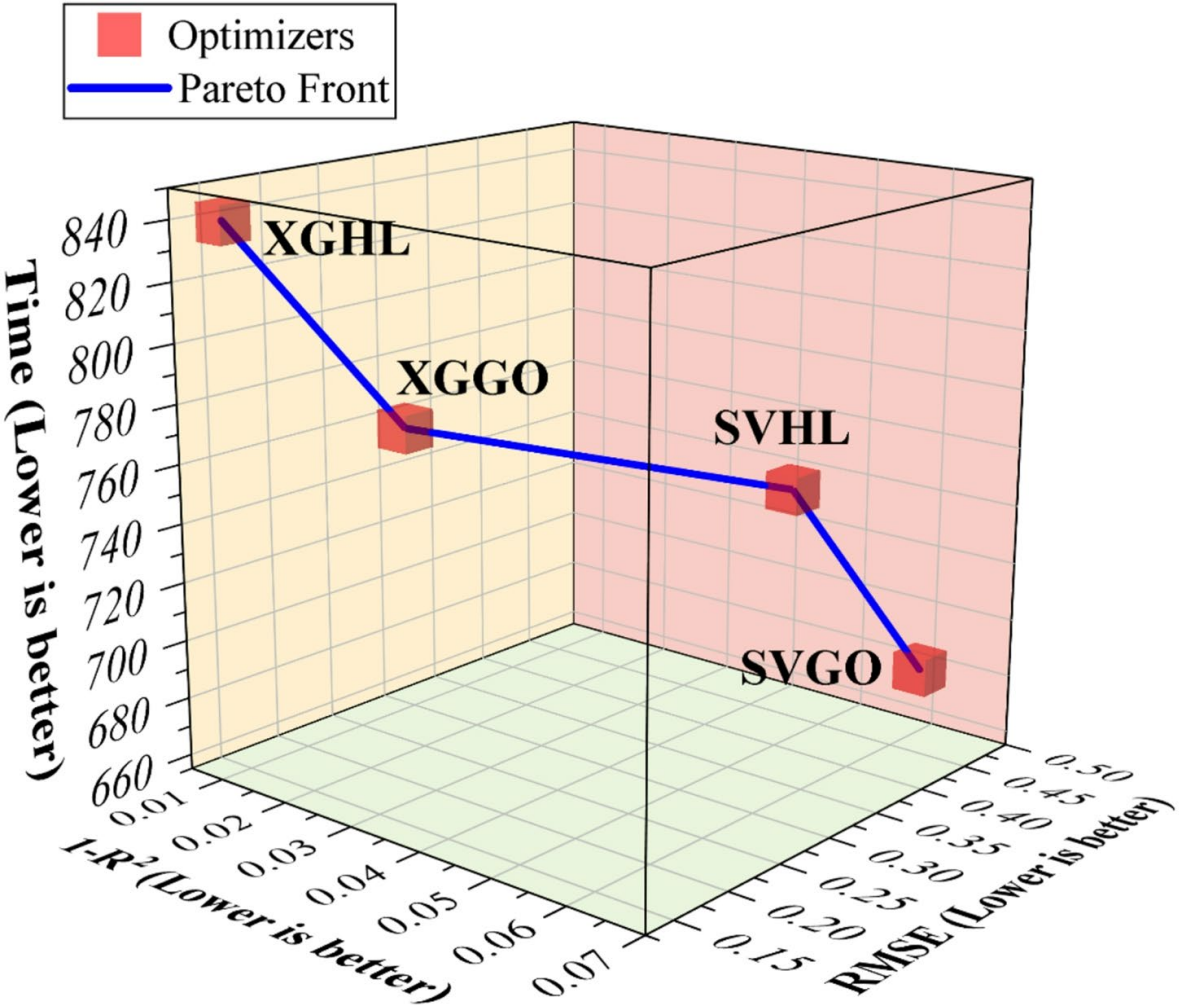
The 3D scatter Pareto plot showing the distribution and relative importance of variables.

The 3D Pareto plot (Fig. 10) integrates model accuracy (RMSE, 1 – R^2^) and computational runtime, illustrating the tradeoffs among the optimized hybrid models. XGHL achieves the lowest RMSE and highest R^2^, reflecting superior predictive fidelity, but at the cost of the longest runtime (837.8 s). XGGO slightly compromises accuracy for a faster runtime (765.5 s), providing a balanced alternative. SVHL and SVGO exhibit higher errors but require moderately lower computational effort, highlighting scenarios where faster screening may be prioritized. This Pareto-based visualization enables informed model selection according to the relative importance of predictive accuracy versus computational cost.

### Feature analysis

**Fig. 11:**
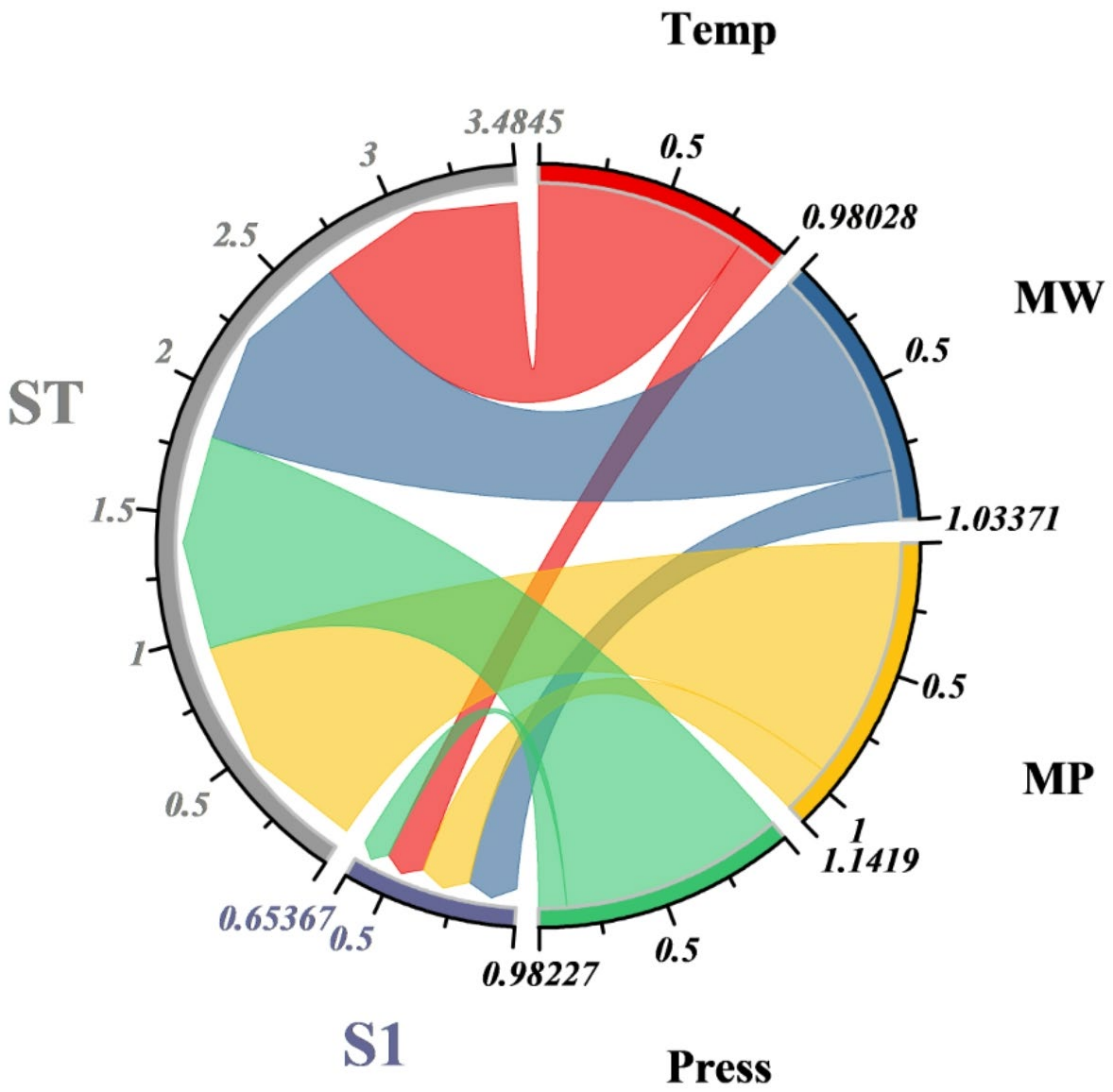
Chord diagram for the FAST sensitivity analysis, showing the relationships and relative contributions among input features.

A detailed exploration of feature contributions was performed using two complementary interpretability techniques: the Feature Attribution Sensitivity Test (FAST) and the Cosine Amplitude Method (CAM). Together, these analyses reveal not only the individual influence of each descriptor but also the interactive relationships among molecular and thermodynamic variables governing solubility in supercritical CO_2_. Fig. 11 presents the FAST chord diagram, which partitions total variance into first-order (S1) and total-order (ST) sensitivity indices. The width of each outer arc represents the individual effect of a feature, while the connecting chords capture higher-order interactions. Pressure emerges as the dominant driver of solubility, displaying the largest combined S1 and ST scores and strong bidirectional chords with molecular weight and melting point. This indicates that pressure not only exerts a direct influence by altering solvent density but also modulates the impact of molecular size and lattice energy on solubility. Molecular weight shows the second-highest sensitivity, reflecting the well-known steric and mass-related constraints on supercritical dissolution. Temperature and melting point exhibit more modest first-order effects but participate in several interaction chords, underscoring their secondary yet synergistic role when coupled with pressure changes.

**Fig. 12:**
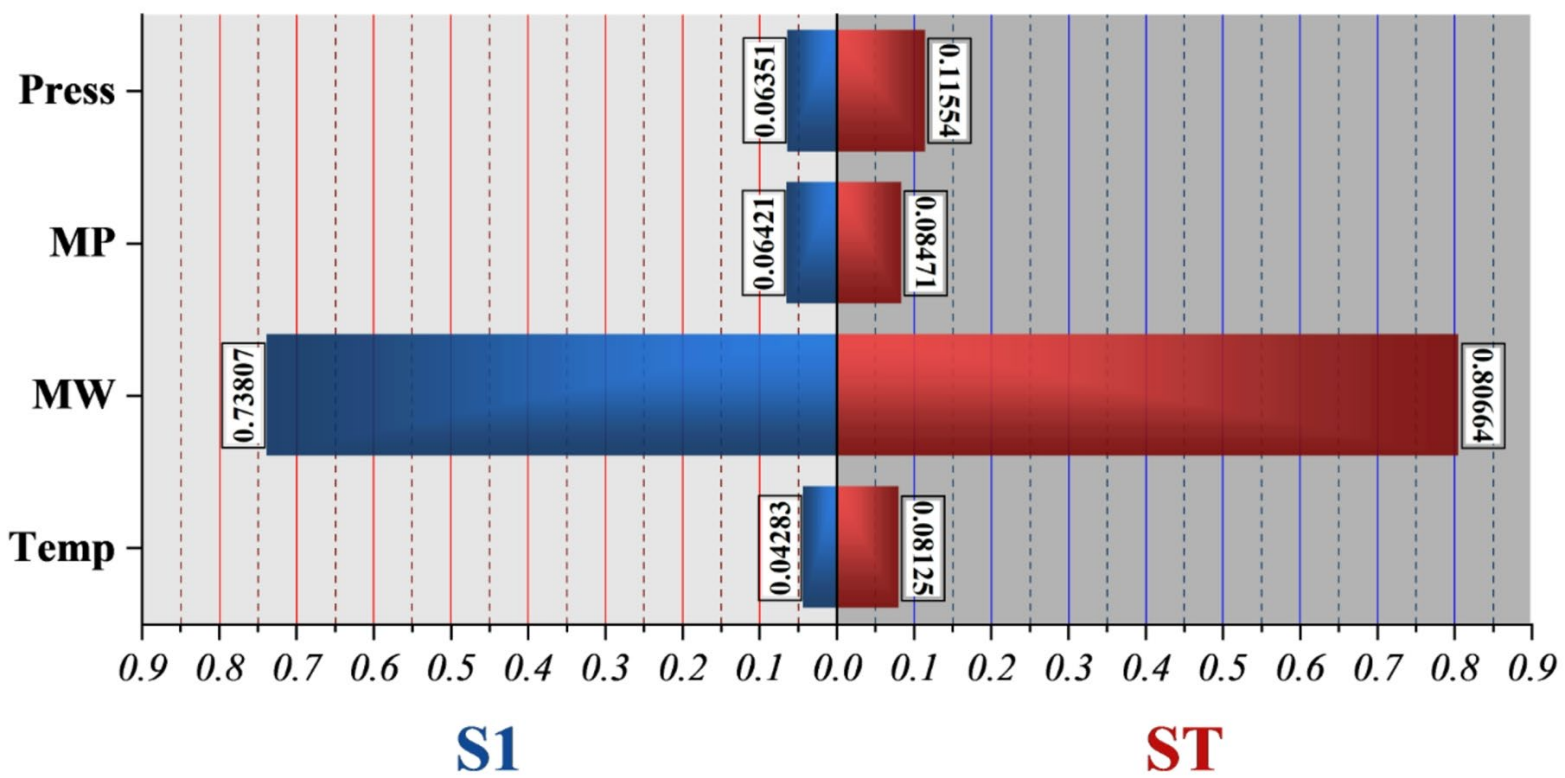
Population pyramid for CAM sensitivity analysis, showing the relative importance of input features.

The CAM population pyramid in Fig. 12 reinforces these findings by separately quantifying the main effects (S1) and total effects (ST). Consistent with the FAST analysis, pressure displays the greatest total influence, followed by molecular weight, confirming that these variables collectively account for the majority of variance in solubility predictions. Interestingly, the CAM method shows a larger total-order contribution for temperature than its first-order effect, suggesting that temperature primarily influences solubility through interactive mechanisms, such as modulating pressure-induced density changes or altering the drug’s vapor pressure. Table 7 shows the obtained values of the CAM and FAST analyses to support the graphical plots.

**Table 7:** Obtained values for the CAM and FAST analyses.

Variables	CAM		FAST	
	S1	ST	S1	ST
Temperature	0.0428	0.0812	0.1544	0.8259
molecular weight	0.7381	0.8066	0.1958	0.8379
melting point	0.0642	0.0847	0.1945	0.9474
Pressure	0.0635	0.1155	0.1090	0.8733

**Fig. 13:**
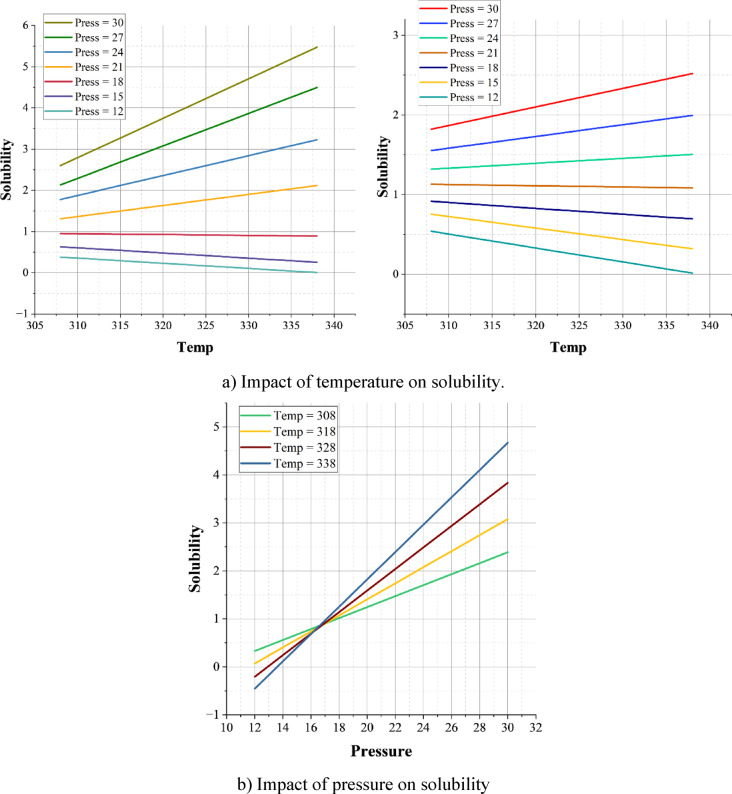

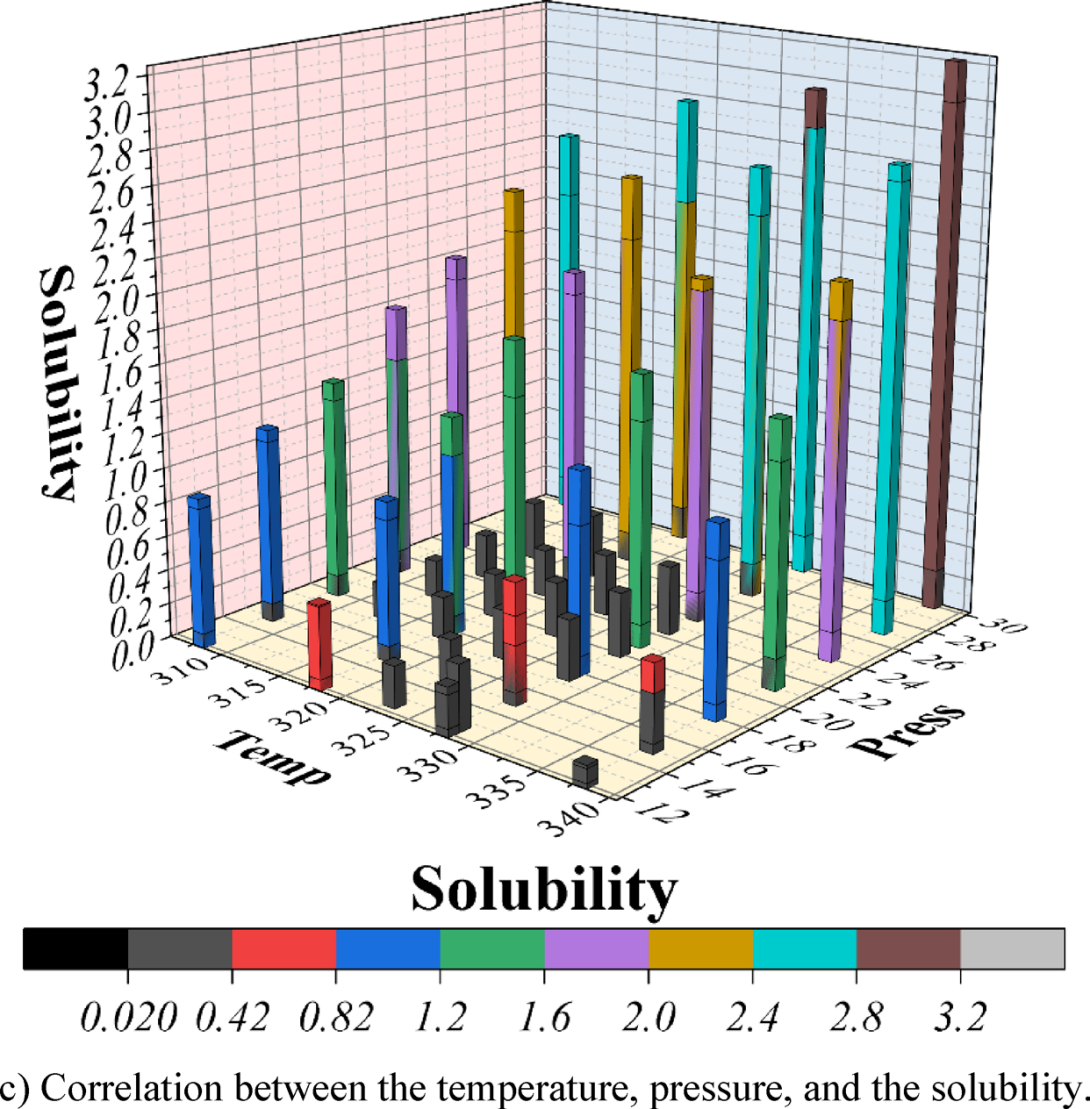
Analysing the impact and relation of the features on solubility using the best model results. (**a**) Impact of temperature on solubility (**b**) Impact of pressure on solubility (**c**) Correlation between the temperature, pressure, and the solubility.

Figure 13 collectively illustrates the coupled effects of temperature and pressure on solubility, highlighting the necessity of incorporating both thermodynamic and molecular descriptors in predictive modeling. In Fig. 13a and b, temperature exhibits a predominantly positive influence on solubility over the investigated pressure range. For example, at 30 MPa, solubility increases from approximately 3.5 g/L at 308 K to about 6 g/L at 338 K, reflecting the enhancement of molecular mobility and the weakening of crystal lattice forces at elevated temperatures. Pressure also exerts a positive effect on solubility at fixed temperature. As shown in Fig. 13b, solubility increases with pressure for all examined temperatures. This behavior is consistent with classical supercritical fluid thermodynamics, in which increasing pressure raises CO_2_ density and, consequently, solvent strength. The higher solvent density enhances dispersive and quadrupolar interactions between CO_2_ and drug molecules, promoting dissolution. From a process-design perspective, this indicates that high-pressure operation in supercritical CO_2_ systems can be exploited to improve solubility, provided that temperature is simultaneously optimized to balance density and vapor-pressure effects.

The 3D correlation plot (Fig. 13c) provides an integrated view, illustrating how temperature amplifies pressure sensitivity. At lower temperatures (308–318 K), pressure exerts only a mild suppressive effect on solubility. In contrast, at higher temperatures (328–338 K), the decline is more pronounced, revealing a nonlinear coupling between these two variables. This dual dependence explains why many purely QSAR-based solubility models fail in real-world applications—they omit thermodynamic conditions that critically shape dissolution.

The purpose of the interpretability analysis in this work is not to propose new thermodynamic mechanisms for solubility in supercritical CO_2_, which are well established in the literature, but to verify that the machine learning framework reproduces physically consistent structure–property relationships across chemically distinct drug systems. By combining CAM-based attribution with variance-based FAST sensitivity, the analysis distinguishes between variables that dominate intrinsic molecular resistance to dissolution (e.g., molecular weight and melting point) and those that primarily control solvent power through interaction effects (e.g., temperature and pressure). This dual perspective extends beyond simple correlation analysis by showing that pressure exerts influence mainly through interaction with temperature and melting point, while molecular weight acts as a dominant local driver of volatility and diffusivity. The agreement between model-derived importance patterns and established thermodynamic expectations indicates that the trained models are not merely interpolating numerically but encode mechanistically meaningful dependencies. Such consistency is critical for deployment in formulation-oriented screening, where physically implausible extrapolations would undermine trust even if statistical accuracy is high.

#### Feature importance to physical chemistry

The feature-importance results obtained from CAM and FAST should be interpreted in light of their complementary methodological perspectives. CAM provides model-specific attribution by quantifying how individual inputs contribute to predictions along learned nonlinear decision paths, and therefore emphasizes molecular weight as the dominant descriptor (S₁ = 0.738), reflecting its strong influence on volatility, diffusivity, and cohesive energy at the molecular scale. In contrast, FAST is a global variance-based method that decomposes output variability into first-order and total-order contributions across the entire input domain. Its higher total-order sensitivity for melting point and temperature indicates that these variables exert a substantial influence primarily through interaction effects with pressure rather than as isolated drivers. Pressure remains a key control variable due to its direct impact on CO_2_ density and solvent power; however, the combined results demonstrate that solubility is governed by a multiscale hierarchy of effects: molecular weight dominates intrinsic molecular resistance to dissolution, melting point reflects lattice stability, and temperature modulates vapor-pressure and density-driven mechanisms in interaction with pressure. Accordingly, the apparent differences between CAM and FAST rankings do not represent inconsistency but rather reveal complementary aspects of solubility control, with CAM highlighting local model sensitivity and FAST capturing global interaction-dominated variability.

### Practical implications

The findings of this study have several practical implications for pharmaceutical research and development. By improving predictive accuracy for drug solubility, the proposed hybrid models, particularly XGHL, demonstrate the potential to support early-stage formulation screening. In practical terms, reducing RMSE from approximately 0.35 (plain XGB) to 0.21 (XGHL) lowers the probability of misclassifying poorly soluble compounds as viable candidates, thereby decreasing the likelihood of advancing weak formulations into resource-intensive experimental stages. Consequently, the models can be employed as pre-screening tools to rank compounds across temperature and pressure ranges prior to extensive laboratory testing, offering potential reductions in material usage and trial-and-error experimentation. Several limitations should be considered when interpreting these results. First, the dataset contains approximately 250 samples across seven drugs. Although these compounds span diverse therapeutic classes, the overall sample size remains limited, and broader molecular coverage would be required to further enhance generalizability. Second, the incorporation of bio-inspired optimization substantially improves predictive performance but introduces significant computational overhead. The reported runtimes for XGB and SVR (0.71 s and 0.63 s, respectively) correspond only to single-pass training using fixed hyperparameters, whereas the runtimes for hybrid models (680–840 s) include the complete hyperparameter optimization procedure. This distinction indicates that the increased computational cost primarily reflects the global search process rather than the intrinsic training complexity of the base learners. Such runtimes are acceptable for offline preclinical analysis, where accuracy is prioritized, but they may be unsuitable for time-critical or real-time applications.

Third, the current feature set is limited to basic thermodynamic and molecular descriptors, including temperature, pressure, molecular weight, and melting point. The inclusion of additional physicochemical descriptors, such as logP, polar surface area, and hydrogen-bonding capacity, could provide a richer chemical representation and further improve predictive performance. All runtime measurements reported in Table 8 were obtained under a standardized computational environment to ensure reproducibility and fair comparison. Training and evaluation were conducted on a workstation equipped with an Intel Core i7-12700 H CPU (14 cores, 2.3–4.7 GHz) and 16 GB RAM without GPU acceleration. The operating system was Windows 11 (64-bit), and the models were implemented in Python 3.10 using scikit-learn 1.4, XGBoost 1.7, and custom implementations of the GGO and HLOA optimizers.

**Table 8:** Computational runtime of each model. Reported values for XGB and SVR correspond to single-pass training with fixed hyperparameters, whereas values for hybrid models include the full bio-inspired hyperparameter optimization procedure.

Models	Runtime
XGHL	837.8 s
XGGO	765.5 s
XGB	0.71 s
SVHL	743.4 s
SVGO	681.5 s
SVR	0.63 s

### Comparison with the published study

Table 9 summarizes recent ML approaches for predicting drug solubility in supercritical CO_2_, highlighting their modeling strategies, input variables, and optimization or interpretability techniques. While prior studies such as Roosta et al. (2025) and others have shown excellent predictive accuracy (e.g., R^2^ ≈ 0.99), they often lack either interpretability or multi-optimizer tuning. In contrast, our proposed framework combines a hybrid ensemble (XGBR + SVR + XGSV) with two bio-inspired optimizers (GGO and HLOA) and dual interpretability methods (FAST and CAM). This integration achieves high accuracy (R^2^ = 0.975, RMSE = 0.21) and, importantly, gives insight into how molecular features and process variables jointly govern solubility. While several recent studies report higher R^2^ values using ensemble or deep learning models, their analyses are typically limited to specific compounds or narrow datasets and focus predominantly on predictive performance. In contrast, the present study integrates heterogeneous experimental data, multiple model classes, and multi-objective optimization into a single comparative framework. The results demonstrate that model performance and stability depend strongly on optimizer–model interaction, and that physically interpretable feature hierarchies can be obtained consistently across diverse drug systems. Accordingly, the contribution is not defined by marginal accuracy gains but by the ability to analyze robustness, generalization, and mechanistic consistency within a single modeling strategy.

**Table 9:** Comparison between the present and published studies.

Study	Modeling approach	Input features	Optimization / interpretability	Key performance
Roosta et al.^20^	LS-SVM, MLP	temp, pressure, SC-CO_2_ density, atomic contribution descriptors	N/A	R^2^ ≈ 0.99 ARD% ≈ 7.20%
Alsaab and Shirzian^46^	Ensemble: XGBR + LGBR + CatBoost	temperature, pressure, molecular weight, melting point	Bio-inspired (Artificial Protozoa Optimizer, Hippopotamus Optimization); interpretability via SHAP + FAST	R^2^ = 0.992 RMSE = 0.0888
Najmi et al.^47^	Stacked ML: Extra Trees + GB + Random Forest	solvent & solute properties, operating conditions	Sensitivity & trend analysis	R^2^ = 0.9981 AARD = 8.62%
Li et al.^48^	Deep Neural Network (DNN), Random Forest	σ-profile descriptors, molecular weight, pressure, temperature, density	Feature reduction of σ-profiles	DNN: R^2^ = 0.9911 RF: R^2^ = 0.9892
Hagbani et al.^49^	MLP, K-NN, Gaussian Process Regression (GPR)	pressure, temperature	Standard ML; interpretability not deeply explored	R^2^ > 0.99 for some models
Wang et al.^50^	Polynomial Regression, Gaussian Process Regression, other ML	temp, pressure	Comparative ML analysis; evaluation of bias–variance tradeoff	Strong fit and process-design guidance
Present study	Ensemble: XGBR + SVR + Hybrid (XGSV)	Molecular descriptors, process parameters (e.g., temperature, pressure)	Bio-inspired optimizers: GGO, HLOA; Feature importance analysis using FAST and CAM	R^2^ = 0.975 RMSE = 0.210

### Suggestion for future study

Future research can extend the current framework by incorporating transfer learning strategies to enhance model generalization across a broader range of pharmaceutical compounds and supercritical solvent systems. Because experimental solubility datasets in SC-CO_2_ are often small and compound-specific, transfer learning could enable models trained on one drug family to be adapted efficiently to another through partial parameter reuse, reduced training requirements, and enhanced extrapolation to low-data regimes. Another promising direction is the development of deep-learning-based hybrid architectures, such as XGBoost–LSTM, CNN–SVR, or GNN–XGB combinations. These models leverage the strengths of tree-based learners in handling tabular physicochemical descriptors while using sequence- or graph-based layers to capture deeper structural patterns in molecular topology or temperature–pressure trajectories. For example, an XGBoost–LSTM hybrid model could capture the temporal or sequential dependence of solubility behavior across varying operational conditions, resulting in smooth prediction surfaces and enhanced sensitivity to nonlinear interactions. Similarly, graph neural networks (GNNs) could incorporate atomic connectivity information directly, enhancing the model’s ability to generalize from one molecular structure to structurally related analogues. Integrating these advanced learning paradigms with physics-informed constraints such as density–pressure relationships or solubility crossover temperatures may further improve reliability and interpretability.

Although the dataset encompasses multiple therapeutic categories to enhance overall model generalizability, the proposed framework can also be interpreted in the context of specific drug classes. Compounds within the same class (e.g., non-steroidal anti-inflammatory drugs or antibiotics) typically share common physicochemical features, such as molecular weight range, functional groups, and polarity. Because the model relies on molecular descriptors and thermodynamic variables rather than categorical labels, it implicitly learns these shared characteristics. Consequently, predictions for drugs belonging to the same class are expected to exhibit similar solubility trends under comparable pressure and temperature conditions. In future work, class-specific subsets of the dataset could be used to construct specialized models for particular therapeutic groups, enabling finer resolution of structure–solubility relationships within each class. This dual capability general modeling across diverse compounds and focused modeling within specific drug families demonstrates the flexibility of the proposed approach.

Such developments would help bridge the gap between data-driven prediction and mechanistic understanding, ultimately creating robust, transferable solubility models applicable to a wider range of drug compounds, cosolvent systems, and supercritical processing technologies.

### Limitations

Although the proposed framework demonstrates strong predictive accuracy, several quantitative limitations should be acknowledged. The dataset used in this study was compiled from six independent experimental sources reported by different research groups, with measurements obtained under varying laboratory conditions, instruments, and CO_2_ purification grades. While this multi-source compilation increases chemical diversity, the dataset remains limited to six drug compounds and a pressure range of 8–32 MPa, which may restrict generalizability to highly polar, thermolabile, or structurally complex molecules not represented in the current sample. Furthermore, the dataset does not include measurements from multiple geographic regions or industrial-scale facilities, limiting the demographic and institutional diversity of the underlying experiments. As a result, potential variations arising from laboratory-to-laboratory calibration differences, operator practices, or CO_2_ purity grades cannot be fully captured or quantified.

In addition, despite the inclusion of multiple drugs and a broad range of operating conditions, the size of the available dataset remains limited (252 samples), and the distribution of samples across individual compounds is uneven. Such imbalance may introduce bias toward compounds with greater representation and can reduce predictive reliability for underrepresented drugs. Consequently, while the proposed models demonstrate strong predictive performance within the explored chemical and thermodynamic space, caution should be exercised when extrapolating predictions to structurally dissimilar compounds or poorly represented drug classes. Future studies should aim to expand the dataset with additional experimental measurements and to achieve more uniform coverage across drug families, which would further enhance the robustness and generalizability of the modeling framework.

These factors collectively indicate that, while the ML models perform robustly within the studied domain, their predictive reliability may decrease when extrapolated to new molecular classes, extreme operating conditions, or untested experimental environments.

## Conclusion

This study developed and benchmarked advanced ML frameworks for predicting drug solubility under varying thermodynamic conditions, with a particular focus on integrating optimization algorithms into XGBoost and SVR architectures. The results consistently demonstrated that hybrid XGBoost models, especially XGHL, achieved the highest accuracy and robustness, outperforming both baseline and ensemble configurations. The XGHL model reduced RMSE by nearly 40% compared to plain XGB, while maintaining excellent alignment with experimental distributions, underscoring its ability to faithfully reproduce the complex nonlinear interplay between molecular weight, melting point, temperature, and pressure. From a pharmaceutical perspective, these gains translate into tangible benefits. More reliable solubility predictions can reduce the frequency of costly wet-lab failures, accelerate compound screening, and provide early guidance in formulation design. The Pareto-based multi-objective optimization further demonstrated how predictive accuracy and computational cost can be balanced, offering practical flexibility: XGHL can serve as the model of choice for high-stakes regulatory or late-stage development, whereas XGGO provides a faster, resource-efficient alternative for exploratory screening. The feature sensitivity analyses reinforced pressure and molecular weight as dominant drivers of solubility, while also revealing important interactive effects involving temperature and melting point. These insights emphasize the necessity of including thermodynamic descriptors alongside molecular properties, bridging the gap between purely QSAR-based approaches and experimentally grounded predictive frameworks. Despite these advances, the study also highlighted limitations. The relatively modest dataset (~ 250 samples across seven drugs) constrains generalizability, while reliance on a limited set of molecular descriptors may underrepresent key solvation dynamics. Moreover, although XGHL achieved the strongest predictive performance, this improvement comes with a noticeably higher computational cost (837.8 s), which must be weighed against its accuracy gains when selecting a model for real-world deployment. Furthermore, the computational overhead of hybrid models, although acceptable for preclinical research, may restrict deployment in time-critical or resource-constrained environments.

## Supplementary Information

Below is the link to the electronic supplementary material.

Supplementary Material 1

Supplementary Material 2

## Data Availability

The datasets used and/or analysed during the current study available from the corresponding author on reasonable request.

## Code availability

The machine learning and optimizations code in addition to dataset gathered in this study are provided in the Supplementary Material.
