# Supplementary material for: Accelerating supercritical pharmaceutical formulation via interpretable data-driven prediction of drug solubility
Source: Sci Rep. 2026 Mar 14;16:11006. doi: 10.1038/s41598-026-44161-9 (PMC13043671; doi:10.1038/s41598-026-44161-9)
Supplement: Supplementary file 2 — Supplementary Material 2 [file 41598_2026_44161_MOESM2_ESM.pdf]

A. Developed Models codes used in the study

1. GGO_SVR

```
import numpy as np
import sklearn.svm as sv
from ypstruct import structure
import sklearn.metrics as met


def positions(nvar, lb, ub, npop):
    Positions = []
    for i in range(npop):
        Positions.append(np.random.uniform(lb, ub, nvar))
    return Positions


def cost_function(pop, X_tr, X_te, Y_tr, Y_te):
    net = sv.SVR(C=pop[0], epsilon=pop[1]/1000, gamma=pop[2])

    net.fit(X_tr, Y_tr)
    preds_tr = net.predict(X_tr)
    preds_te = net.predict(X_te)
    y = np.hstack([Y_tr, Y_te])
    preds = np.hstack([preds_tr, preds_te])
    MSE = met.mean_squared_error(y, preds)
    RMSE = np.sqrt(MSE)
    POP = structure(cost=RMSE, pre=preds, net=net)
    return POP.cost, POP.pre, POP.net



# %% DATA LOADING
XX = np.loadtxt('input.txt')
Y = np.loadtxt('target.txt')
size_Y = len(Y)

size_tr = round(size_Y*0.8)
size_te = size_Y-size_tr

X_tr = XX[:size_tr]
X_te = XX[size_tr:]
Y_tr = Y[:size_tr]
Y_te = Y[size_tr:]

# Greylag Goose Optimization
# function [best_solution, best_fitness] = ggo(obj_func, num_variables, num_agents,
max_iter, lb, ub)
num_variables = 3
lb = 1              # % Lower limit for variables
```

```
ub = 999             # % Upper limit for variables
num_agents = 50
max_iter = 200

POP_empty = structure(cost=None, pre=None)
POP = POP_empty*num_agents
new_POP = structure(cost=None, pre=None)
conv = np.zeros(max_iter)

# % Initialize population
population = positions(num_variables, lb, ub, num_agents)
fitness = np.zeros(num_agents)

# % Evaluate fitness of each agent
for i in range(num_agents):
    # fitness[i] = obj_func(population(i, :));
    fitness[i], POP[i].pre = cost_function(population[i], X_tr, X_te, Y_tr, Y_te)
    POP[i].Cost = fitness[i]

# % Main loop
for iter1 in range(max_iter):
    # % Update position and fitness of each agent
    for i in range(num_agents):
        # % Determine the best agent in the flock
        best_agent_index = np.argmin(fitness)

        # % Generate a new solution by combining exploration and exploitation
        new_solution = population[i] + np.random.rand(num_variables) *
(population[best_agent_index] - population[i])

        # % Clip new solution to ensure it stays within bounds
        # new_solution = max(min(new_solution, ub), lb);
        for j in range(num_variables):
            new_solution[j] = max(new_solution[j], lb)
            new_solution[j] = min(new_solution[j], ub)

        # % Evaluate fitness of the new solution
        # new_fitness = obj_func(new_solution);
        new_fitness, new_POP.pre = cost_function(new_solution, X_tr, X_te, Y_tr, Y_te)
        new_POP.Cost = new_fitness

        # % Update if the new solution is better
        if new_fitness < fitness[i]:
            population[i] = new_solution
            fitness[i] = new_fitness
            POP[i].pre = new_POP.pre


# % Find the best solution in the final population
    best_index = np.argmin(fitness)
```

```
    best_fitness = min(fitness)
    conv[iter1] = best_fitness
    print(iter1,best_fitness)
    best_solution = population[best_index]
    best_pre = POP[best_index].pre
```

2. GGO_XGBR

```
import numpy as np
import xgboost as xgb
from ypstruct import structure
import sklearn.metrics as met


def positions(nvar, lb, ub, npop):
    Positions = []
    for i in range(npop):
        Positions.append(np.random.uniform(lb, ub, nvar))
    return Positions


def cost_function(pop, X_tr, X_te, Y_tr, Y_te):
    net = xgb.XGBRegressor(n_estimators=int(pop[0]), max_depth=int(pop[1]),
                           learning_rate=(pop[2]/1000.0),colsample_bytree=pop[3]/1000,

subsample=pop[4]/1000,reg_alpha=pop[5]/1000,reg_lambda=pop[6]/1000,
                           objective='reg:squarederror')
    net.fit(X_tr, Y_tr)
    preds_tr = net.predict(X_tr)
    preds_te = net.predict(X_te)
    y = np.hstack([Y_tr, Y_te])
    preds = np.hstack([preds_tr, preds_te])
    MSE = met.mean_squared_error(y, preds)
    RMSE = np.sqrt(MSE)
    POP = structure(cost=RMSE, pre=preds, net=net)
    return POP.cost, POP.pre, POP.net



# %% DATA LOADING
XX = np.loadtxt('input.txt')
Y = np.loadtxt('target.txt')
size_Y = len(Y)

size_tr = round(size_Y*0.8)
size_te = size_Y-size_tr

X_tr = XX[:size_tr]
X_te = XX[size_tr:]
Y_tr = Y[:size_tr]
Y_te = Y[size_tr:]
```

```
# Greylag Goose Optimization
# function [best_solution, best_fitness] = ggo(obj_func, num_variables, num_agents,
max_iter, lb, ub)
num_variables = 7
lb = 1                  # % Lower limit for variables
ub = 999                # % Upper limit for variables
num_agents = 50
max_iter = 200

POP_empty = structure(cost=None, pre=None)
POP = POP_empty*num_agents
new_POP = structure(cost=None, pre=None)
conv = np.zeros(max_iter)

# % Initialize population
population = positions(num_variables, lb, ub, num_agents)
fitness = np.zeros(num_agents)

# % Evaluate fitness of each agent
for i in range(num_agents):
    # fitness[i] = obj_func(population(i, :));
    fitness[i], POP[i].pre = cost_function(population[i], X_tr, X_te, Y_tr, Y_te)
    POP[i].Cost = fitness[i]

# % Main loop
for iter1 in range(max_iter):
    # % Update position and fitness of each agent
    for i in range(num_agents):
        # % Determine the best agent in the flock
        best_agent_index = np.argmin(fitness)

        # % Generate a new solution by combining exploration and exploitation
        new_solution = population[i] + np.random.rand(num_variables) *
(population[best_agent_index] - population[i])

        # % Clip new solution to ensure it stays within bounds
        # new_solution = max(min(new_solution, ub), lb);
        for j in range(num_variables):
            new_solution[j] = max(new_solution[j], lb)
            new_solution[j] = min(new_solution[j], ub)

        # % Evaluate fitness of the new solution
        # new_fitness = obj_func(new_solution);
        new_fitness, new_POP.pre = cost_function(new_solution, X_tr, X_te, Y_tr, Y_te)
        new_POP.Cost = new_fitness

        # % Update if the new solution is better
        if new_fitness < fitness[i]:
            population[i] = new_solution
```

```
        fitness[i] = new_fitness
        POP[i].pre = new_POP.pre


# % Find the best solution in the final population
    best_index = np.argmin(fitness)
    best_fitness = min(fitness)
    conv[iter1] = best_fitness
    print(iter1,best_fitness)
    best_solution = population[best_index]
    best_pre = POP[best_index].pre
```

3. HLO_SVR

```
import numpy as np
import sklearn.svm as sv
from ypstruct import structure
import sklearn.metrics as met

def positions(nvar, lb, ub, npop):
    Positions = []
    for i in range(npop):
        Positions.append(np.random.randint(lb, ub, nvar))
    return Positions

def cost_function(pop, X_tr, X_te, Y_tr, Y_te):
    net = sv.SVR(C=pop[0], epsilon=pop[1]/1000, gamma=pop[2])

    net.fit(X_tr, Y_tr)
    preds_tr = net.predict(X_tr)
    preds_te = net.predict(X_te)
    y = np.hstack([Y_tr, Y_te])
    preds = np.hstack([preds_tr, preds_te])
    MSE = met.mean_squared_error(y, preds)
    RMSE = np.sqrt(MSE)
    POP = structure(cost=RMSE, pre=preds, Net=net)
    return POP.cost, POP.pre , POP.Net


def alpha_melanophore(fit,vMin,vMax):
    o = np.zeros(len(fit))
    for i in range(len(fit)):
        o[i] = (vMax-fit[i])/(vMax-vMin)
    return o



def R(NP):
    band = 1
    while band:
        r1 = round(1+ (NP-1)*np.random.rand())
```

```
        r2 = round(1+ (NP-1)*np.random.rand())
        r3 = round(1+ (NP-1)*np.random.rand())
        r4 = round(1+ (NP-1)*np.random.rand())
        if all([r1 != r2 , r2 != r3 , r1 != r3 , r4 != r3 , r4 != r2 , r1 != r4]):
            band= 0
    return r1,r2,r3,r4


def get_color(colorPalette):
     band = 1
     while band:
         c1 = colorPalette[np.random.randint(0, 29)]
         c2 = colorPalette[np.random.randint(0, 29)]
         if (c1 != c2):
             band= 0
     return c1,c2

def get_binary():
    if np.random.rand() < 0.5:
        val = 0
    else:
        val = 1
    return val

def mimicry(Xbest, X, Max_iter, SearchAgents_no,t):
    colorPalette = [0, 0.00015992, 0.001571596, 0.001945436, 0.002349794, 0.00353364,
0.0038906191, 0.003906191, 0.199218762, 0.19999693, 0.247058824, 0.39999392,
0.401556397, 0.401559436, 0.498039216, 0.498046845, 0.499992341, 0.49999997,
0.601556397, 0.8, 0.900000447, 0.996093809, 0.996109009, 0.996872008, 0.998039245,
0.998046875, 0.998431444, 0.999984801, 0.999992371, 1]
    Delta = 2
    r1, r2, r3, r4 = R(SearchAgents_no-1)
    c1, c2 = get_color(colorPalette)
    o = Xbest +  (Delta-Delta*t/Max_iter) * (c1*((np.sin(X[r1])-np.cos(X[r2])) - ((-
1)**get_binary()) * c2*np.cos(X[r3])-np.sin(X[r4])))
    return o


def shoot_blood_stream(Xbest, X, Max_iter,t):
    g = 0.009807    # % 9.807 m/s2   a kilometros    =>  0.009807 km/s2
    epsilon= 1E-6
    Vo = 1  # %1E-2;
    Alpha = np.pi/2
    o = ( Vo * np.cos(Alpha*t/Max_iter)+epsilon) * Xbest + (Vo * np.sin(Alpha-
Alpha*t/Max_iter)-g+epsilon) * X
    return o

def cauchy_rand(m,c):
    cauchy = c*np.tan(np.pi*(np.random.rand()-0.5)) + m
    return cauchy
```

```
def random_walk(Xbest,X):
  e =  cauchy_rand(0,1)
  walk = -1 + 2 * np.random.rand()  # % -1 < d < 1
  o = Xbest + walk*(0.5-e)*X
  return o


def skin_darkening_or_lightening(Xbest,  X, SearchAgents_no):
    darkening = [0.0, 0.4046661]
    lightening = [0.5440510 , 1.0]
    dark1= darkening[0] + (darkening [1] - darkening[0])* np.random.rand()
    dark2= darkening[0] + (darkening [1] - darkening[0])* np.random.rand()
    light1= lightening[0] + (lightening[1]-lightening[0])* np.random.rand()
    light2= lightening[0] + (lightening[1]-lightening[0])* np.random.rand()
    r1, r2, r3, r4 = R(SearchAgents_no-1)
    if get_binary():
         o = Xbest + light1*np.sin((X[r1]-X[r2])/2) - ((-1)**get_binary()) *
light2*np.sin((X[r3]-X[r4])/2)
    else:
         o = Xbest + dark1*np.sin((X[r1]-X[r2])/2) - ((-1)**get_binary()) *
dark2*np.sin((X[r3]-X[r4])/2);

    return o

def remplace_SearchAgent(Xbest, X, SearchAgents_no):
    band = 2
    while band:
          r1= round((SearchAgents_no-1)*np.random.rand())
          r2= round((SearchAgents_no-1)*np.random.rand())
          if r1 != r2:
             band= 0;

    o=  Xbest + (X[r1]-((-1)**get_binary())*X[r2])/2

    return o

# %  ******************************************
# %  HLOA: Horned Lizard Optimization Algorithm
# %  Developed in MATLAB R2018a(9.4)                         %
# %  Author/Programmer:  Dr. Hernan Peraza-Vazquez                        %
# %  email: hperaza@ipn.mx                          %
# %  Telegram: @CodebugMx                                   %
# %  Paper: A Novel Metaheuristic Inspired by Horned Lizard Defense Tactics %
# %  Artificial Intelligence Review - Springer (2024)
# %  Doi:         10.1007/s10462-023-10653-7
#
%____________________________________________________________________________
__%
```

```
# function
[vMin,theBestVct,Convergence_curve]=HLOA(SearchAgents_no,Max_iter,lb,ub,dim,fobj)

# %% DATA LOADING
X=np.loadtxt('input.txt')
Y=np.loadtxt('target.txt')
size_Y = len(Y)
size_tr = round(size_Y*0.8)
size_te = size_Y-size_tr

X_tr = X[:size_tr]
X_te = X[size_tr:]
Y_tr = Y[:size_tr]
Y_te = Y[size_tr:]

Y_tr = np.array(Y_tr).reshape(size_tr)
Y_te = np.array(Y_te).reshape(size_te)

dim = 3
lb = 1
ub = 999
SearchAgents_no = 50
Max_iter = 200


POP_empty = structure(cost=None, pre=None, net=None)
POP = POP_empty*SearchAgents_no
New_POP = POP_empty
bestSol = structure(cost=None, pre=None, net=None, positions=None)
Fitness = np.zeros(SearchAgents_no)
v = np.zeros(SearchAgents_no*dim)
v = np.array(v).reshape(SearchAgents_no,dim)

Positions = positions(dim,lb,ub,SearchAgents_no)


for i in range(SearchAgents_no):
    # Fitness(i)=fobj(Positions(i,:)); % get fitness
    Fitness[i], POP[i].pre, POP[i].net = cost_function(Positions[i], X_tr, X_te, Y_tr, Y_te)
    POP[i].Cost = Fitness[i]

minIdx = np.argmin(Fitness)
vMin = min(Fitness)   # % the min fitness value vMin and the position minIdx
theBestVct= Positions[minIdx]  # % the best vector

bestSol.position= theBestVct
bestSol.Cost= vMin
bestSol.pre = POP[minIdx].pre

maxIdx = np.argmax(Fitness)
```

```
vMax = max(Fitness)   # % the max fitness value vMax and the position maxIdx
Convergence_curve = np.zeros(Max_iter)
Convergence_curve[0] = vMin
alphaMelanophore= alpha_melanophore(Fitness,vMin,vMax)


# % Main
for t in range(1,Max_iter):
    for r in range(SearchAgents_no):
        if 0.5 < np.random.rand():   # % se mimetiza
             v[r] = mimicry(theBestVct, Positions, Max_iter, SearchAgents_no, t)
        else:
            if(np.mod(t,2)):
                v[r] = shoot_blood_stream(theBestVct, Positions[r], Max_iter, t)
            else:
                v[r] = random_walk(theBestVct,Positions[r])

        Positions[maxIdx] = skin_darkening_or_lightening(theBestVct, Positions,
SearchAgents_no)
        if alphaMelanophore[r] <= 0.3:
           v[r] = remplace_SearchAgent(theBestVct, Positions,SearchAgents_no)

        # %-------------------------------------------------------------------
        # % Return back the search agents that go beyond the boundaries of the search space
        # v[r] = checkBoundaries(v[r], lb, ub)
        for j in range(dim):
            v[r][j] = max(v[r][j],lb)
            v[r][j] = min(v[r][j],ub)

        # % Evaluate new solutions
        # Fnew= fobj(v(r,:));
        Fnew, New_POP.pre, New_POP.net = cost_function(v[r], X_tr, X_te, Y_tr, Y_te)
        New_POP.Cost = Fnew

        # % Update if the solution improves
        if Fnew <= Fitness[r]:
            Positions[r] = v[r]
            Fitness[r] = Fnew
            POP[r].pre = New_POP.pre

        if Fnew <= vMin:
            theBestVct= v[r]
            vMin= Fnew

            bestSol.position= theBestVct
            bestSol.Cost= vMin
            bestSol.pre = New_POP.pre

    # %update max and alpha-melanophore
    maxIdx = np.argmax(Fitness)
```

```
    vMax = max(Fitness)   # % the max fitness value vMax and the position maxIdx
    alphaMelanophore = alpha_melanophore(Fitness,vMin,vMax)
    Convergence_curve[t] = vMin
    print(t,vMin)
```

4. HLO_XGBR

```
import numpy as np
import xgboost as xgb
from ypstruct import structure
import sklearn.metrics as met

def positions(nvar, lb, ub, npop):
   Positions = []
   for i in range(npop):
      Positions.append(np.random.randint(lb, ub, nvar))
   return Positions

def cost_function(pop, X_tr, X_te, Y_tr, Y_te):
   net = xgb.XGBRegressor(n_estimators=int(pop[0]), max_depth=int(pop[1]),
                    learning_rate=(pop[2]/1000.0),colsample_bytree=pop[3]/1000,

subsample=pop[4]/1000,reg_alpha=pop[5]/1000,reg_lambda=pop[6]/1000,
                    objective='reg:squarederror')
   net.fit(X_tr, Y_tr)
   preds_tr = net.predict(X_tr)
   preds_te = net.predict(X_te)
   y = np.hstack([Y_tr, Y_te])
   preds = np.hstack([preds_tr, preds_te])
   MSE = met.mean_squared_error(y, preds)
   RMSE = np.sqrt(MSE)
   POP = structure(cost=RMSE, pre=preds, Net=net)
   return POP.cost, POP.pre , POP.Net


def alpha_melanophore(fit,vMin,vMax):
   o = np.zeros(len(fit))
   for i in range(len(fit)):
       o[i] = (vMax-fit[i])/(vMax-vMin)
   return o



def R(NP):
   band = 1
   while band:
         r1 = round(1+ (NP-1)*np.random.rand())
         r2 = round(1+ (NP-1)*np.random.rand())
         r3 = round(1+ (NP-1)*np.random.rand())
         r4 = round(1+ (NP-1)*np.random.rand())
```

```
        if all([r1 != r2 , r2 != r3 , r1 != r3 , r4 != r3 , r4 != r2 , r1 != r4]):
            band= 0
    return r1,r2,r3,r4


def get_color(colorPalette):
     band = 1
     while band:
          c1 = colorPalette[np.random.randint(0, 29)]
          c2 = colorPalette[np.random.randint(0, 29)]
          if (c1 != c2):
             band= 0
     return c1,c2

def get_binary():
    if np.random.rand() < 0.5:
        val = 0
    else:
        val = 1
    return val

def mimicry(Xbest, X, Max_iter, SearchAgents_no,t):
    colorPalette = [0, 0.00015992, 0.001571596, 0.001945436, 0.002349794, 0.00353364,
0.0038906191, 0.003906191, 0.199218762, 0.19999693, 0.247058824, 0.39999392,
0.401556397, 0.401559436, 0.498039216, 0.498046845, 0.499992341, 0.49999997,
0.601556397, 0.8, 0.900000447, 0.996093809, 0.996109009, 0.996872008, 0.998039245,
0.998046875, 0.998431444, 0.999984801, 0.999992371, 1]
    Delta = 2
    r1, r2, r3, r4 = R(SearchAgents_no-1)
    c1, c2 = get_color(colorPalette)
    o = Xbest +  (Delta-Delta*t/Max_iter) * (c1*((np.sin(X[r1])-np.cos(X[r2])) - ((-
1)**get_binary()) * c2*np.cos(X[r3])-np.sin(X[r4])))
    return o



def shoot_blood_stream(Xbest, X, Max_iter,t):
    g = 0.009807    # % 9.807 m/s2   a kilometros    =>  0.009807 km/s2
    epsilon= 1E-6
    Vo = 1  # %1E-2;
    Alpha = np.pi/2
    o = ( Vo * np.cos(Alpha*t/Max_iter)+epsilon) * Xbest + (Vo * np.sin(Alpha-
Alpha*t/Max_iter)-g+epsilon) * X
    return o

def cauchy_rand(m,c):
    cauchy = c*np.tan(np.pi*(np.random.rand()-0.5)) + m
    return cauchy


def random_walk(Xbest,X):
```

```
  e =  cauchy_rand(0,1)
  walk = -1 + 2 * np.random.rand()  # % -1 < d < 1
  o = Xbest + walk*(0.5-e)*X
  return o


def skin_darkening_or_lightening(Xbest,  X, SearchAgents_no):
    darkening = [0.0, 0.4046661]
    lightening = [0.5440510 , 1.0]
    dark1= darkening[0] + (darkening [1] - darkening[0])* np.random.rand()
    dark2= darkening[0] + (darkening [1] - darkening[0])* np.random.rand()
    light1= lightening[0] + (lightening[1]-lightening[0])* np.random.rand()
    light2= lightening[0] + (lightening[1]-lightening[0])* np.random.rand()
    r1, r2, r3, r4 = R(SearchAgents_no-1)
    if get_binary():
        o = Xbest + light1*np.sin((X[r1]-X[r2])/2) - ((-1)**get_binary()) *
light2*np.sin((X[r3]-X[r4])/2)
    else:
        o = Xbest + dark1*np.sin((X[r1]-X[r2])/2) - ((-1)**get_binary()) *
dark2*np.sin((X[r3]-X[r4])/2);

    return o

def remplace_SearchAgent(Xbest, X, SearchAgents_no):
    band = 2
    while band:
         r1= round((SearchAgents_no-1)*np.random.rand())
         r2= round((SearchAgents_no-1)*np.random.rand())
         if r1 != r2:
            band= 0;

    o=  Xbest + (X[r1]-((-1)**get_binary())*X[r2])/2

    return o

# %  *******************************************
# %  HLOA: Horned Lizard Optimization Algorithm
# %  Developed in MATLAB R2018a(9.4)                          %
# %  Author/Programmer:  Dr. Hernan Peraza-Vazquez                          %
# %  email: hperaza@ipn.mx                          %
# %  Telegram: @CodebugMx                                   %
# %  Paper: A Novel Metaheuristic Inspired by Horned Lizard Defense Tactics %
# %  Artificial Intelligence Review - Springer (2024)
# %  Doi:        10.1007/s10462-023-10653-7
#
%____________________________________________________________________________
__%
# function
[vMin,theBestVct,Convergence_curve]=HLOA(SearchAgents_no,Max_iter,lb,ub,dim,fobj)
```

```
# %% DATA LOADING
X=np.loadtxt('input.txt')
Y=np.loadtxt('target.txt')
size_Y = len(Y)
size_tr = round(size_Y*0.8)
size_te = size_Y-size_tr

X_tr = X[:size_tr]
X_te = X[size_tr:]
Y_tr = Y[:size_tr]
Y_te = Y[size_tr:]

Y_tr = np.array(Y_tr).reshape(size_tr)
Y_te = np.array(Y_te).reshape(size_te)

dim = 7
lb = 1
ub = 999
SearchAgents_no = 50
Max_iter = 200


POP_empty = structure(cost=None, pre=None, net=None)
POP = POP_empty*SearchAgents_no
New_POP = POP_empty
bestSol = structure(cost=None, pre=None, net=None, positions=None)
Fitness = np.zeros(SearchAgents_no)
v = np.zeros(SearchAgents_no*dim)
v = np.array(v).reshape(SearchAgents_no,dim)

Positions = positions(dim,lb,ub,SearchAgents_no)


for i in range(SearchAgents_no):
    # Fitness(i)=fobj(Positions(i,:)); % get fitness
    Fitness[i], POP[i].pre, POP[i].net = cost_function(Positions[i], X_tr, X_te, Y_tr, Y_te)
    POP[i].Cost = Fitness[i]

minIdx = np.argmin(Fitness)
vMin = min(Fitness)   # % the min fitness value vMin and the position minIdx
theBestVct= Positions[minIdx]  # % the best vector

bestSol.position= theBestVct
bestSol.Cost= vMin
bestSol.pre = POP[minIdx].pre

maxIdx = np.argmax(Fitness)
vMax = max(Fitness)   # % the max fitness value vMax and the position maxIdx
Convergence_curve = np.zeros(Max_iter)
Convergence_curve[0] = vMin
```

```
alphaMelanophore= alpha_melanophore(Fitness,vMin,vMax)


# % Main
for t in range(1,Max_iter):
    for r in range(SearchAgents_no):
        if 0.5 < np.random.rand():   # % se mimetiza
             v[r] = mimicry(theBestVct, Positions, Max_iter, SearchAgents_no, t)
        else:
            if(np.mod(t,2)):
                v[r] = shoot_blood_stream(theBestVct, Positions[r], Max_iter, t)
            else:
                v[r] = random_walk(theBestVct,Positions[r])

        Positions[maxIdx] = skin_darkening_or_lightening(theBestVct, Positions,
SearchAgents_no)
        if alphaMelanophore[r] <= 0.3:
           v[r] = remplace_SearchAgent(theBestVct, Positions,SearchAgents_no)

        # %-------------------------------------------------------------------
        # % Return back the search agents that go beyond the boundaries of the search space
        # v[r] = checkBoundaries(v[r], lb, ub)
        for j in range(dim):
            v[r][j] = max(v[r][j],lb)
            v[r][j] = min(v[r][j],ub)

        # % Evaluate new solutions
        # Fnew= fobj(v(r,:));
        Fnew, New_POP.pre, New_POP.net = cost_function(v[r], X_tr, X_te, Y_tr, Y_te)
        New_POP.Cost = Fnew

        # % Update if the solution improves
        if Fnew <= Fitness[r]:
            Positions[r] = v[r]
            Fitness[r] = Fnew
            POP[r].pre = New_POP.pre

        if Fnew <= vMin:
            theBestVct= v[r]
            vMin= Fnew

            bestSol.position= theBestVct
            bestSol.Cost= vMin
            bestSol.pre = New_POP.pre

    # %update max and alpha-melanophore
    maxIdx = np.argmax(Fitness)
    vMax = max(Fitness)   # % the max fitness value vMax and the position maxIdx
    alphaMelanophore = alpha_melanophore(Fitness,vMin,vMax)
    Convergence_curve[t] = vMin
```

```
print(t,vMin)
```

B. Dataset used in this study

Temperature (K)	molecular weight (g/mol)	melting point (C)	Pressure (MPa)	Solubility (g/l) x 10
318	586.18	146	30	1.94
308	157.1	190	12	0.53
338	926.09	155	21	0.77
308	157.1	190	30	5.13
318	385.37	236	27	0.413
338	586.18	146	24	3.2
318	157.1	190	18	1.3
318	385.37	236	30	0.493
308	385.37	236	27	0.346
318	157.1	190	27	5.41
338	157.1	190	24	5.6
308	337.45	163.5	15	1.06
318	586.18	146	27	1.57
328	157.1	190	27	6.58
328	393.44	230	12	0.074
318	586.18	146	27	1.57
308	157.1	190	24	3.42
328	586.18	146	24	2.12
318	157.1	190	27	5.41
338	926.09	155	30	1.73
328	315.71	239	12	0.2
338	393.44	230	12	0.045
328	586.18	146	12	0.07
308	337.45	163.5	30	3.835
338	157.1	190	12	0.03
318	385.37	236	18	0.166
318	157.1	190	15	0.8
328	393.44	230	30	1.584
318	586.18	146	18	0.58
308	926.09	155	27	1.45
338	393.44	230	30	1.68
318	926.09	155	21	1.02
338	337.45	163.5	21	2.177
318	157.1	190	12	0.37
338	393.44	230	24	1.049

328	157.1	190	21	3.21
328	393.44	230	18	0.368
318	157.1	190	30	6.48
328	586.18	146	27	2.94
308	586.18	146	15	0.24
328	586.18	146	24	2.12
318	157.1	190	30	6.48
328	315.71	239	21	1.91
338	385.37	236	30	0.665
338	157.1	190	24	5.6
338	315.71	239	21	1.83
318	926.09	155	24	1.23
328	385.37	236	27	0.473
338	157.1	190	15	0.37
338	157.1	190	18	1.32
318	393.44	230	27	1.259
338	586.18	146	12	0.04
338	586.18	146	15	0.16
318	315.71	239	30	3.76
308	385.37	236	12	0.042
308	337.45	163.5	24	2.377
318	157.1	190	18	1.3
308	926.09	155	18	0.92
328	157.1	190	12	0.08
308	586.18	146	24	0.61
308	586.18	146	30	0.88
308	385.37	236	21	0.216
308	385.37	236	15	0.097
318	385.37	236	24	0.34
328	157.1	190	18	1.39
328	157.1	190	12	0.08
308	385.37	236	30	0.42
318	157.1	190	24	4.29
338	337.45	163.5	24	3.189
318	337.45	163.5	21	1.895
308	315.71	239	15	1.41
318	337.45	163.5	27	3.445
338	385.37	236	15	0.083
308	157.1	190	21	2.31
308	157.1	190	24	3.42
318	393.44	230	21	0.73
308	586.18	146	12	0.13

328	315.71	239	27	3.3
308	586.18	146	12	0.13
318	586.18	146	24	1.22
308	586.18	146	27	0.74
308	337.45	163.5	21	1.857
318	337.45	163.5	15	0.746
338	385.37	236	21	0.336
328	337.45	163.5	12	0.12
338	586.18	146	18	0.77
308	337.45	163.5	12	0.77
338	157.1	190	21	3.92
308	315.71	239	12	1.09
338	157.1	190	15	0.37
338	337.45	163.5	30	5.153
308	315.71	239	30	3.07
318	926.09	155	12	0.27
338	157.1	190	12	0.03
328	586.18	146	18	0.76
328	393.44	230	27	1.237
318	157.1	190	21	2.72
338	337.45	163.5	12	0.046
328	157.1	190	15	0.6
308	393.44	230	30	1.289
308	393.44	230	12	0.279
308	393.44	230	15	0.395
338	393.44	230	18	0.26
328	393.44	230	24	1.032
338	315.71	239	15	0.56
328	157.1	190	21	3.21
308	393.44	230	27	1.075
308	926.09	155	21	1.15
318	157.1	190	15	0.8
308	586.18	146	18	0.36
318	315.71	239	21	2.01
328	385.37	236	21	0.277
308	393.44	230	18	0.609
328	157.1	190	18	1.39
328	157.1	190	30	7.65
338	157.1	190	21	3.92
328	315.71	239	24	2.52
318	586.18	146	12	0.1
328	337.45	163.5	30	4.714

338	926.09	155	18	0.45
318	393.44	230	18	0.443
328	586.18	146	27	2.94
328	926.09	155	24	1.15
328	157.1	190	15	0.6
318	157.1	190	12	0.37
318	926.09	155	18	0.75
328	586.18	146	30	3.83
318	586.18	146	21	0.89
318	586.18	146	18	0.58
328	337.45	163.5	18	1.325
338	586.18	146	18	0.77
308	385.37	236	24	0.27
328	586.18	146	18	0.76
338	315.71	239	24	2.56
328	337.45	163.5	24	2.742
308	337.45	163.5	18	1.553
308	926.09	155	30	1.61
308	315.71	239	24	2.35
308	586.18	146	15	0.24
308	926.09	155	15	0.69
318	393.44	230	24	1.022
338	337.45	163.5	27	4.294
308	586.18	146	27	0.74
308	157.1	190	27	4.09
338	385.37	236	18	0.21
338	926.09	155	12	0.03
308	586.18	146	30	0.88
328	385.37	236	24	0.384
328	586.18	146	21	1.38
338	393.44	230	27	1.292
338	157.1	190	27	7.57
328	315.71	239	30	4.19
338	157.1	190	27	7.57
308	157.1	190	12	0.53
308	157.1	190	30	5.13
338	586.18	146	24	3.2
328	385.37	236	12	0.028
328	337.45	163.5	27	3.908
338	385.37	236	24	0.433
338	385.37	236	27	0.562
338	315.71	239	27	3.39

308	385.37	236	18	0.144
328	315.71	239	15	0.83
328	385.37	236	18	0.18
328	926.09	155	21	0.89
308	315.71	239	18	1.66
328	926.09	155	30	1.71
308	315.71	239	21	2.03
308	393.44	230	24	0.918
308	315.71	239	27	2.76
308	337.45	163.5	27	3.017
338	586.18	146	27	4.73
338	586.18	146	30	6.12
308	586.18	146	21	0.48
338	337.45	163.5	15	0.329
308	157.1	190	15	0.87
308	586.18	146	24	0.61
328	393.44	230	21	0.673
338	586.18	146	27	4.73
328	926.09	155	15	0.37
318	385.37	236	12	0.034
328	586.18	146	21	1.38
318	586.18	146	21	0.89
338	926.09	155	27	1.53
318	315.71	239	27	2.95
318	315.71	239	18	1.54
328	393.44	230	15	0.175
328	157.1	190	27	6.58
328	337.45	163.5	21	2
318	586.18	146	12	0.1
328	586.18	146	15	0.2
328	385.37	236	30	0.551
338	926.09	155	15	0.22
308	586.18	146	18	0.36
328	157.1	190	30	7.65
318	385.37	236	15	0.094
338	586.18	146	12	0.04
328	586.18	146	15	0.2
308	157.1	190	18	1.44
318	385.37	236	21	0.237
318	926.09	155	15	0.53
308	157.1	190	18	1.44
328	926.09	155	18	0.64

318	926.09	155	27	1.48
338	315.71	239	12	0.11
318	586.18	146	30	1.94
328	315.71	239	18	1.39
338	157.1	190	30	9.05
318	315.71	239	12	0.69
328	586.18	146	12	0.07
338	157.1	190	18	1.32
338	586.18	146	15	0.16
308	157.1	190	27	4.09
318	315.71	239	24	2.44
318	315.71	239	15	1.13
328	157.1	190	24	4.75
318	586.18	146	24	1.22
318	157.1	190	24	4.29
318	586.18	146	15	0.22
318	157.1	190	21	2.72
308	926.09	155	24	1.28
318	337.45	163.5	24	2.557
308	393.44	230	21	0.764
338	157.1	190	30	9.05
318	393.44	230	15	0.296
318	926.09	155	30	1.69
328	385.37	236	15	0.09
328	586.18	146	30	3.83
338	315.71	239	30	4.22
318	337.45	163.5	12	0.351
338	385.37	236	12	0.019
338	315.71	239	18	1.15
328	157.1	190	24	4.75
338	586.18	146	30	6.12
318	586.18	146	15	0.22
318	393.44	230	30	1.494
328	926.09	155	12	0.12
308	586.18	146	21	0.48
308	157.1	190	21	2.31
338	393.44	230	21	0.591
338	393.44	230	15	0.119
318	393.44	230	12	0.161
338	337.45	163.5	18	1.23
318	337.45	163.5	30	4.358
318	337.45	163.5	18	1.399

308	926.09	155	12	0.45
328	337.45	163.5	15	0.542
338	926.09	155	24	1.11
338	586.18	146	21	1.81
338	586.18	146	21	1.81
308	157.1	190	15	0.87
328	926.09	155	27	1.53
